# From individual to relational: a scientometric mapping of emotion regulation in adolescence

**DOI:** 10.3389/fpsyg.2026.1965549

**Published:** 2026-10-08

**Authors:** Gloria Simoncini, Francesca Borghesi, Giulia Ballarotto, Lavinia Barone, Ilaria M. A. Benzi, Claudia Greco, Laura Parolin, Patrizia Velotti, Pietro Cipresso

**Affiliations:** 1 Department of Theoretical and Applied Sciences (DiSTA), eCampus University, Novedrate, Italy; 2 Department of Psychology, University of Turin, Turin, Italy; 3 Department of Dynamic and Clinical Psychology and Health Studies, Sapienza University of Rome, Rome, Italy; 4 Department of Brain and Behavioral Sciences, University of Pavia, Pavia, Italy; 5 Department of Psychology, University of Milano-Bicocca, Milan, Italy

**Keywords:** adolescence, emotion regulation, science mapping, scientometric analysis, youth

## Abstract

**Introduction:**

The study of emotion regulation in adolescence has undergone significant expansion, generating a complex and only partially integrated body of knowledge.

**Methods:**

This study offers a comprehensive scientometric mapping of the literature, drawing on 10,865 documents indexed in the Web of Science Core Collection (1988–2026) and combining performance indicators with science-mapping techniques via the bibliometrix R package. Beyond describing the field’s structure, this study examines its evolution through a relational lens, considering how interpersonal processes such as family context, attachment, and peer dynamics are positioned within the intellectual and conceptual organization of the field.

**Results:**

Performance analysis indicates a field approaching maturity, having reached roughly 75% of its estimated saturation, with annual production estimated to peak around 2023. The findings suggest that, alongside the growing prominence of clinical and transdiagnostic perspectives, developmental and relational foundations have remained structurally visible throughout the evolution of the domain. Intellectually, the findings delineate a progressively reorganized knowledge base, moving from early developmental, coping-, and attachment-based frameworks toward process-oriented and transdiagnostic perspectives. Conceptually, the contemporary structure is strongly organized around the intersection between emotion regulation, depression, and anxiety, while developmental, neurodevelopmental, relational, and psychosocial themes remain represented in more specialized areas of the field. Socially, the results reveal a collaborative structure organized around several cohesive author and institutional communities, connected through broader inter-institutional and international links.

**Discussion:**

Collectively, these patterns suggest that adolescent emotion regulation research has moved toward greater theoretical and structural integration while retaining differentiated research traditions and emerging areas of development.

## 1 Introduction

Emotion regulation (ER) sits at the core of adaptive functioning and mental health, comprising implicit and explicit skills used to monitor, evaluate, and modify emotional responses in accordance with one’s own goals (Silvers, 2022; Young et al., 2019; Velotti et al., 2013). Within the broader life-cycle, adolescence emerges as a developmental phase of exceptional dynamism and vulnerability, marked by rapid neurobiological and social transformations that intensify emotional reactivity (Fombouchet et al., 2023; Silvers, 2022). During this transition, a fundamental shift occurs: from the external support provided by caregivers toward greater independence and the increasing reliance on internal strategies for affective management (Casey et al., 2010).

Emotional dysregulation is now widely recognized as a transdiagnostic factor implicated across internalizing, externalizing, and neurodevelopmental conditions (Aldao et al., 2016; te Brinke et al., 2021; England-Mason, 2020). In fact, ER is not merely a potential risk or protective factor, but a dynamic process that evolves in response to context, one that helps explain the comorbidity and heterotypic continuity of psychopathologies over time (Rogier et al., 2024; Aldao et al., 2016). From this vantage point, recent research has begun to probe specific clinical outcomes, demonstrating how regulatory difficulties are crucial to understanding trajectories of resilience or risk for non-suicidal self-injury (NSSI), (Escofet-Colet et al., 2025).

The developmental trajectories of ER are profoundly shaped by social influences. While parental emotional socialization remains highly relevant, more recent research (Azevedo et al., 2025; Ruan et al., 2023) highlights the need to move beyond the analysis of isolated individual strategies, instead exploring the interdependence and interactive nature of parental responses through network analysis. At the same time, adolescence marks a period of social reorientation in which peers’ responses to emotional expressions (PREE) become pivotal in shaping individual regulatory capacities (Delios et al., 2023).

Alongside this growing complexity, contemporary research has increasingly moved beyond the evaluation of single “adaptive” or “maladaptive” strategies toward constructs such as emotion regulation flexibility and regulatory repertoires (Fombouchet et al., 2023; Grommisch et al., 2020; Haag et al., 2024). Methodological developments, including network-based approaches, latent profile analysis, and ecological momentary assessment, have further enabled the study of regulatory processes as dynamic and context-dependent phenomena in adolescents’ everyday lives (Grommisch et al., 2020; Ruan et al., 2023; Thunnissen et al., 2021).

Yet this explosion of empirical contributions, fueled by the exponential increase in the number of researchers and the ease of access to digital information (Aria et al., 2020), for all that it reflects the vitality of the domain, has produced research streams that are enormously voluminous and deeply fragmented (Aria and Cuccurullo, 2017). The literature on emotion regulation has, in fact, been described since its inception as “a topic in search of definition” (Silvers, 2022; Thompson, 1994), characterized by a persistent lack of consensus on its internal structure, which has inevitably slowed the field’s progress (Compas et al., 2017).

The proliferation of often poorly coordinated models (Compas et al., 2017), combined with inconsistent operational definitions, which frequently confuse regulation strategies with underlying abilities (Fombouchet et al., 2023) or which vary significantly across age groups (Haag et al., 2024), makes traditional narrative reviews insufficient for obtaining a coherent *big picture* of the entire scientific landscape. Qualitative approaches are limited by the sheer volume of data and the heterogeneity of study designs and populations analyzed, making it difficult to synthesize results (Aldao et al., 2016).

The present study was designed precisely to fill this gap, providing a systematic, transparent, and reproducible mapping of the intellectual, conceptual, and social architecture of emotional regulation in adolescence. In doing so, this study adopts a specific interpretive lens. Although emotion regulation has traditionally been theorized as an internal, individual process, a growing body of work reframes it as socially scaffolded and relationally enacted, particularly in adolescence, a period defined by a tension between the developmental push toward autonomy and a persistent reliance on relational sources of regulation. This relational view resonates with the broader notion of emotion regulation as a process in which caregivers and peers actively participate in shaping how adolescents manage affect, rather than as a purely intrapersonal achievement. The present analysis therefore examines not only how the field is structured, but whether its intellectual and conceptual architecture reflects this relational dimension. Through the use of the bibliometrix R package and advanced science mapping techniques (Aria and Cuccurullo, 2017), the research sets out to:

1. Identify the intellectual structure of the field, tracing the intellectual turning points and foundational theoretical anchors that have shaped its development.
2. Examine the conceptual structure, delineating the evolution of themes, from classical developmental models to the more recent transdiagnostic and technological frontiers.
3. Analyze the social structure, uncovering the global collaborative networks that sustain scientific production in this area.

Across these three structural levels, particular attention is paid to where and how interpersonal and relational processes like family context, attachment, and peer dynamics, surface as organizing forces of the field. The analyses provide a broader picture of a research domain that has progressively moved from fragmentation toward greater theoretical and structural integration. Across its intellectual, conceptual, and social dimensions, the literature reveals an increasingly interconnected field in which developmental and clinical perspectives coexist with persistent relational foundations.

## 2 Materials and methods

### 2.1 Study design and bibliometric workflow

To provide a systematic and reproducible overview of the research landscape concerning emotion regulation in adolescence, this study adopts a scientometric approach based on science mapping. Following recent methodological guidance, the analysis was organized as a structured bibliometric workflow encompassing the definition of research objectives, literature search and data collection, data cleaning and preprocessing, selection and application of bibliometric techniques, visualization, and interpretation and reporting (Passas, 2024; Kumar, 2025). This methodology allows for the objective identification of the conceptual, intellectual, and social structures that characterize this scientific domain. The reporting of the present scientometric review was informed by the preliminary BIBLIO guideline for bibliometric reviews (Montazeri et al., 2023). A completed BIBLIO checklist indicating where each reporting item is addressed is provided in the Supplementary Table S7.

### 2.2 Data collection and search strategy

The bibliographic dataset analyzed in the present study was retrieved from the Web of Science Core Collection (WoS), one of the most widely used databases for scientometric research due to its high-quality indexing system and standardized citation records (Falagas et al., 2008). The overall workflow adopted for the construction of the dataset is illustrated in Figure 1.

**Figure 1:**
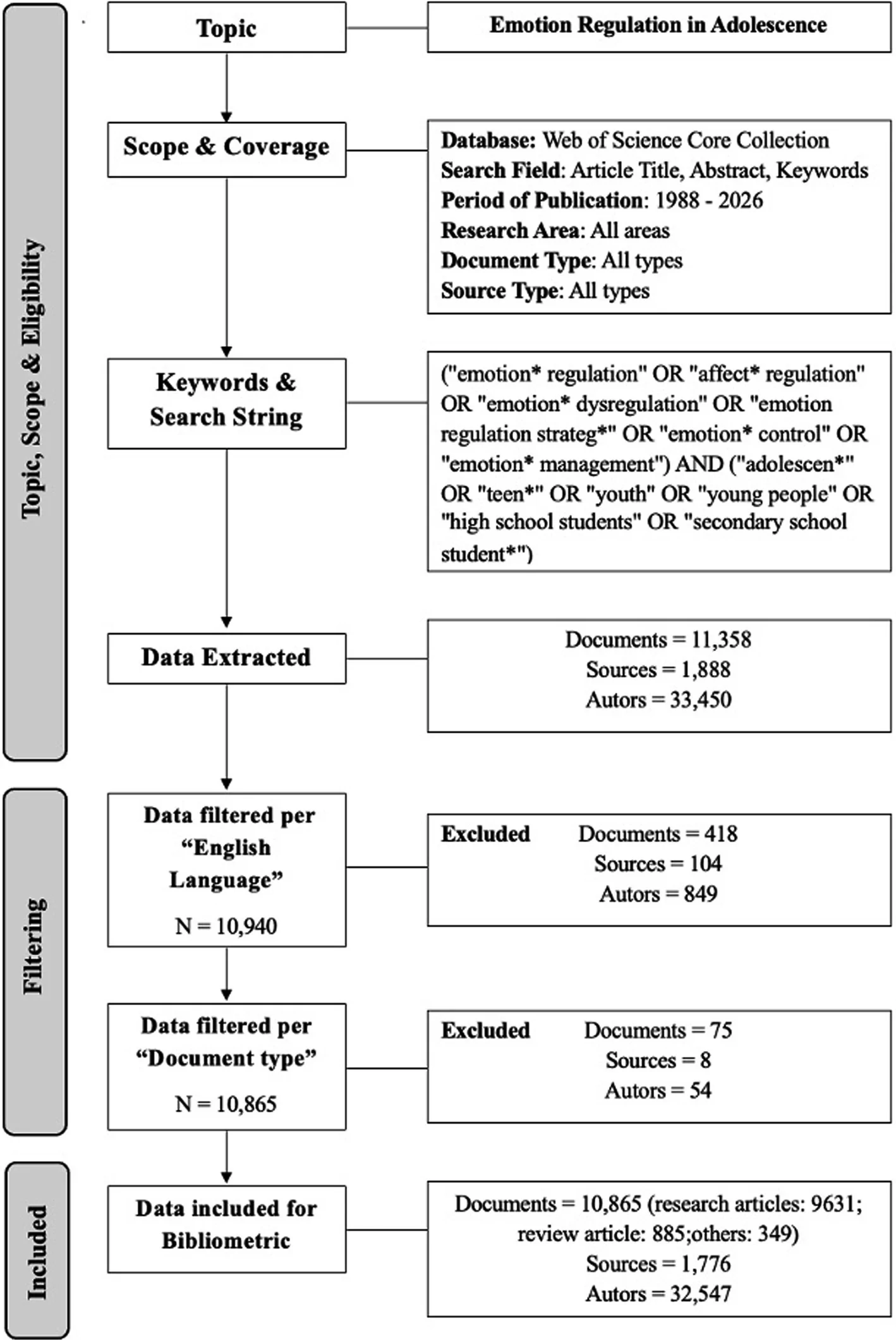
Flow diagram of the dataset selection process used to construct the bibliometric corpus.

To identify the scientific literature on emotion regulation in adolescence, a search query was constructed combining terms related to emotion regulation processes and adolescent populations. The search string used was the following: (“emotion* regulation” OR “affect* regulation” OR “emotion* dysregulation” OR “emotion regulation strateg*” OR “emotion* control” OR “emotion* management”) AND (“adolescen*” OR “teen*” OR “youth” OR “young people” OR “high school students” OR “secondary school student*”).

The population-related terms were intentionally defined broadly to maximize recall, given the variability with which adolescent populations are described across the literature. The query was applied to the Web of Science Core Collection without temporal restrictions in order to capture the full historical development of the field (until February, 17th 2026). The initial search retrieved 11,358 documents, originating from 1,888 sources and authored by 33,450 researchers (see Figure 1).

Several filtering procedures were applied to ensure the consistency of the dataset. First, the search results were restricted to documents written in English, reducing the dataset to 10,940 documents (1,784 sources and 32,601 authors) in order to avoid linguistic heterogeneity that could affect textual and keyword-based analyses (Figure 1). Second, a document type filter was applied to include only specific categories indexed in Web of Science. The following document types were retained in the dataset: *Article; Article Early Access; Article Book Chapter; Article Data Paper; Article Proceedings Paper; Review; Review Early Access; Review Book Chapter; Proceedings Paper; Meeting Abstract; Letter; Letter Early Access; and Book Review*. After applying this filter, the final dataset consisted of 10,865 documents, published across 1,776 sources and authored by 32,547 researchers, which were used for the subsequent bibliometric analyses (Figure 1).

All bibliographic records were exported in plain text format including full metadata (authors, title, abstract, keywords, cited references, and source information), which were required for the subsequent scientometric analyses.

### 2.3 Data analysis

The bibliometric and scientometric analyses were conducted using the R statistical environment, employing the open-source package bibliometrix, which provides a comprehensive framework for quantitative research in bibliometrics and science mapping (Aria and Cuccurullo, 2017, 2026). The package allows the conversion of bibliographic records from Web of Science into structured data frames and supports a wide range of descriptive and network-based analyses commonly used in scientometric studies.

Bibliometric methods can broadly encompass evaluative approaches, which describe characteristics and impact of scholarly output, and relational approaches, which examine relationships among bibliographic units through shared metadata such as citations, keywords, and authors (Ninkov et al., 2022). Following established and current bibliometric frameworks, the analytical strategy of the present study combines performance-oriented indicators with relational science-mapping techniques to examine productivity, impact, and the structural relationships among publications, authors, institutions, and concepts (Zupic and Čater, 2015; Passas, 2024; Kumar, 2025).

#### 2.3.1 Performance analysis: productivity and impact

Performance analysis was conducted to assess the scientific productivity, citation impact, and thematic development of research on emotion regulation in adolescence. This approach relies on descriptive bibliometric indicators derived from the metadata of the retrieved publications.

First, general descriptive statistics of the dataset were examined through the main information table, which summarizes key characteristics of the bibliographic corpus, including the number of documents, authors, sources, citations, and collaboration indicators. In addition, the life cycle of scientific production was analyzed to investigate the temporal evolution of the field and to identify growth patterns in the number of publications over time. The analysis follows the methodology proposed by Meyer et al. (1999) and implemented in the lifeCycle function of the bibliometrix package. The logistic model estimates several parameters describing the evolution of the research field, including the carrying capacity (K), the growth rate, and the inflection point (t0) corrensponding to the period of maximum expansion.

To evaluate the scientific impact of the literature, several citation-based indicators were considered. The temporal pattern of citation impact was also examined through the average annual citation rate of publications according to their year of publication. This indicator provides a time-normalized view of citation performance across publication cohorts; however, values for the most recent years were interpreted cautiously because of the shorter citation windows available to newer publications. In particular, the analysis identified the most relevant sources, highlighting the journals that contribute most extensively to the field. Furthermore, the most locally cited documents and most locally cited references were examined in order to identify the most influential publications within the dataset and the foundational works that have shaped the development of the research domain. Local citation analysis was performed using the localCitations() function implemented in the bibliometrix R-package. Unlike global citation counts (GCS), which capture a publication’s overall impact across the database, local citations (LCS) measure how often a document is cited within the dataset itself, providing a more precise indicator of intellectual influence within the specific research domain. Locally cited references were extracted and normalized using the citations() and tableTag(Tag = “CR”) functions.

Finally, the thematic orientation and evolution of the field were explored through textual analyses of authors’ keywords. Prior to the keyword-based analyses, author keywords were harmonized using a manually curated synonym list implemented in bibliometrix, given the need to account for synonyms and alternative labels when using automated keyword-based bibliometric approaches (Greener, 2022). To minimize researcher-driven conceptual aggregation, normalization was restricted to terms showing strict lexical equivalence, including singular and plural forms, spelling and orthographic variants, hyphenation variants, direct lexical synonyms, and unambiguous abbreviations and their expanded forms. Conceptually related but non-equivalent terms were retained as separate keywords. The complete keyword harmonization dictionary is provided as Supplementary file S2. For the word-cloud analysis only, the most generic search-defining terms directly reflecting the core construct and target population were excluded from the visualization in order to reduce their tautological predominance and allow other thematic patterns in the literature to emerge more clearly. The normalized author-keyword field was then used for the word-cloud, trend-topic, and thematic-map analyses.

#### 2.3.2 Science mapping: conceptual, social and intellectual structures

In addition to descriptive indicators, science-mapping techniques were applied to investigate the research field’s structural organization. Science mapping focuses on identifying relationships among scientific publications and enables the visualization of the conceptual, social, and intellectual structures underlying a research domain.

Trend topic and word cloud analyses (KeywordGrowth() function) were complemented by science-mapping techniques. A deeper understanding of the conceptual structure of the field was obtained through the thematic map, generated from the normalized author-keyword field using the thematicMap() function. This strategic diagram organizes themes according to two key dimensions: centrality, which reflects the relevance of a theme within the broader research field, and density, which indicates the degree of internal development and cohesion of the theme. A keyword co-occurrence network was also constructed to identify the main thematic clusters characterizing the literature.

To explore the field’s social structure, collaboration networks were constructed at different levels of analysis. In particular, an authors’ collaboration network was constructed using the biblioNetwork() function with the parameters *analysis = “collaboration”* and *network = “authors.”* Node size reflects author productivity and edge thickness the strength of collaboration; community-detection algorithms identified cohesive research groups. Furthermore, to explore collaboration patterns at the institutional level, affiliation data were extracted using the metaTagExtraction() function and subsequently used to construct an institutional collaboration network through biblioNetwork(network = “universities”). In this network, nodes represent research institutions and edges indicate co-authored publications between researchers affiliated with different universities. For both collaboration networks, communities were identified using the Louvain community-detection algorithm, corresponding to the default clustering procedure implemented in Biblioshiny.

Finally, to examine the evolution of the intellectual structure of the analyzed domain, a Reference Co-citation Analysis was conducted using the biblioNetwork() function. Grounded in the assumption that jointly cited works share intellectual affinity, this technique reconstructs the field’s knowledge base (Aria et al., 2020).

To capture the dynamic development of the domain, the main dataset was divided into three distinct time intervals through a time-slicing procedure implemented via the timeslice() function (Aria and Cuccurullo, 2017). The complete observation period was partitioned into three consecutive 13-calendar-year intervals: TF1 (1988–2000), TF2 (2001–2013), and TF3 (2014–2026; including records available through February 17, 2026). Equal-duration intervals were adopted to provide a consistent temporal basis for comparing changes in the field’s co-citation structure across successive periods. The use of multiple time slices to trace the development of a research field is an established strategy in science mapping (Aria and Cuccurullo, 2017), and comparable diachronic co-citation studies have employed consecutive equal-length subperiods to examine stability, change, and the emergence of new intellectual configurations over time (White and McCain, 1998).

For each temporal interval, a co-citation matrix was constructed using biblioNetwork with the parameters *analysis = “co-citation”* and *network = “references.”* To reduce network complexity and focus on the most influential intellectual contributions, a filtering procedure was applied by selecting, for each period, the top 50 most frequently cited references through the helper function *extract_top_refs*. Node importance was operationalized through Node Strength (igraph strength function), i.e., the sum of co-citation link weights at each node.

To identify coherent schools of thought or thematic areas, the Louvain algorithm (multi-level modularity optimisation) was applied for community detection using the *cluster_louvain* function. To further characterize the role of seminal works, centrality metrics (Betweenness and Closeness; Supplementary Table S1) were computed using the *betweenness* and *closeness* functions. Betweenness centrality was used to identify works acting as intellectual bridges between clusters, whereas Closeness centrality reflects the proximity of a work to the theoretical core of its reference community, consistent with classical network analysis approaches (Brandes, 2001).

Finally, to ensure visual comparability across the three periods (TF1, TF2, TF3), an advanced diachronic stability strategy was implemented. A shared layout was computed based on the average of the expanded adjacency matrices (via the *expand_matrix* function) across the three periods and processed using the Fruchterman-Reingold algorithm through the *layout_with_fr* function. This ensures that persisting nodes retain similar coordinates across maps, facilitating visual tracking of the field’s evolution; a custom match_cluster_colors function assigned consistent colors to overlapping communities across periods.

To assess the robustness of the network-based findings to document-type heterogeneity, a sensitivity analysis was conducted by excluding Meeting Abstracts and Letters (including Early Access). The reference co-citation networks, author-keyword co-occurrence network, thematic map, and authors’ collaboration network were recomputed using the same analytical parameters as in the main analyses.

## 3 Results

### 3.1 Performance analysis: productivity and impact

#### 3.1.1 Main information

Table 1 summarizes the main descriptive information of the bibliographic dataset analyzed in this study. The final dataset consists of 10,865 documents published between 1988 and 2026, originating from 1,776 different sources, including journals, books, and conference proceedings. Overall, the dataset includes 321,525 cited references, indicating a substantial knowledge base supporting research on emotion regulation in adolescence. The literature exhibits a steady expansion over time, with an annual growth rate of 13.34%, and an average of 27.18 citations per document, suggesting a relevant level of scholarly impact.

**Table 1:** Main information about the bibliographic dataset, including descriptive statistics, document types, authorship indicators, and keyword information for the literature on emotion regulation in adolescence.

Description	Results
Main information about data	
Timespan	1988–2026
Sources (Journals, Books, etc)	1776
Documents	10,865
Annual Growth Rate %	13.34
Average citations per doc	27.18
References	321,525
Documents contents	
Author’s Keywords (DE)	13,400
Authors	
Authors	32,547
Authors of single-authored docs	433
International co-authorships %	21.75
Document types	
Research Articles (article; article, early access; article, book chapter; article, proceedings paper; article, data paper)	9,631
Review Articles (review; review, early access; review, book chapter)	885
Other document types (meeting abstract; proceedings paper; book review; letter; letter, early access; editorial material, book chapter)	349

From a content perspective, the dataset contains 13,400 author keywords (DE), reflecting the conceptual richness and thematic diversity of the field. The scientific production involves 32,547 authors, of whom 433 contributed to single-authored documents, while the vast majority of publications were produced through collaborative research. In addition, 21.75% of the documents involve international co-authorship, indicating a moderate level of cross-country collaboration.

Regarding document types, the dataset is largely dominated by research articles (*n* = 9,631), which represent the primary vehicle for disseminating empirical findings in the field. Review articles account for 885 documents, providing theoretical syntheses of the literature, while a smaller proportion consists of other document types (*n* = 349), including meeting abstracts, proceedings papers, letters, and book reviews.

The temporal pattern of citation impact is illustrated in Figure 2, which reports the average annual citation rate of publications according to their year of publication. The profile shows substantial fluctuations during the earliest years of the field, when the limited number of publications makes annual averages particularly sensitive to the citation performance of individual documents. From the early 2000s onward, citation rates became more stable, with comparatively higher values throughout much of the 2000s, followed by a gradual decline in more recent publication years.

**Figure 2:**
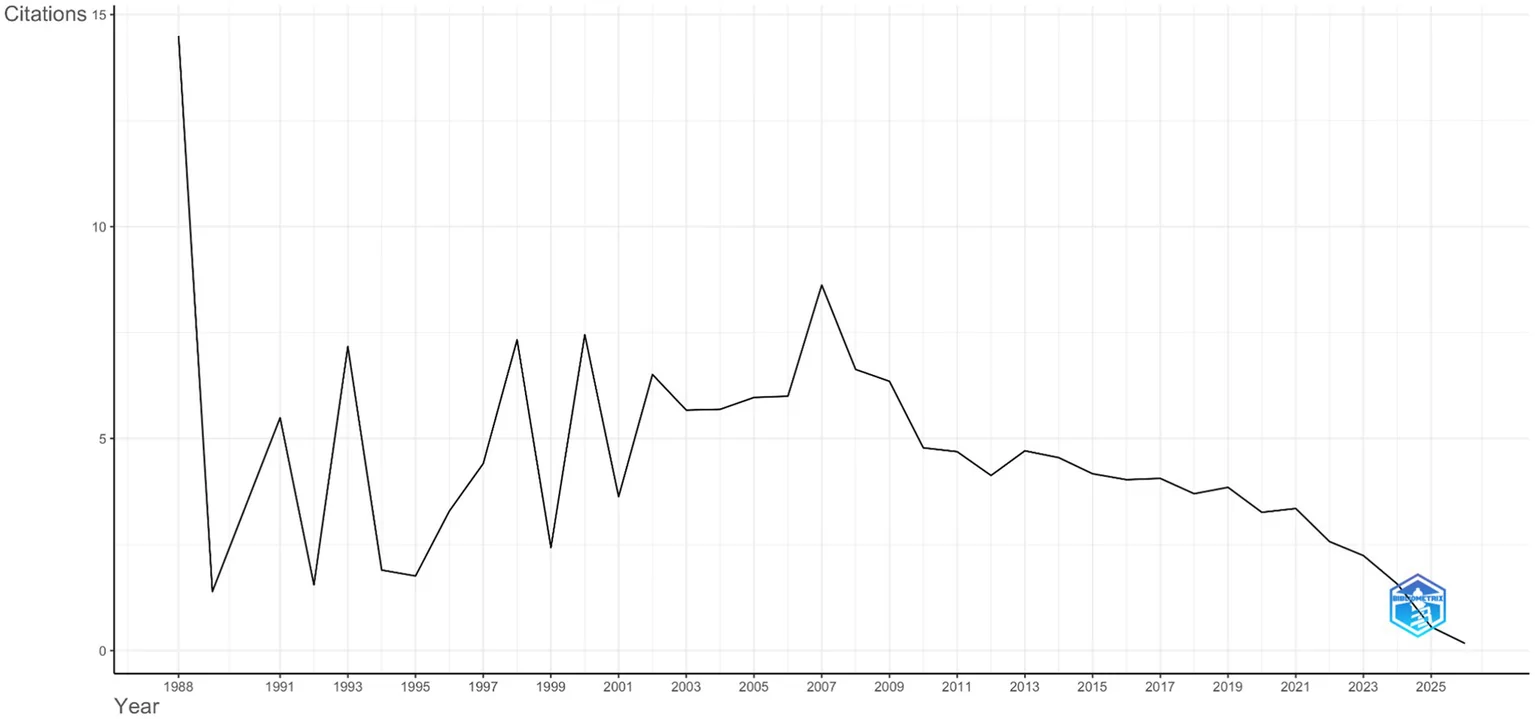
Average citations per year for publications on emotion regulation in adolescence, according to year of publication (1988–2026).

This downward pattern should not be interpreted as evidence of decreasing scientific relevance. More recent publications have had substantially less time to accumulate citations, and citation lag particularly affects the latest years of the observation period. Accordingly, the markedly lower values observed toward the end of the series primarily reflect the shorter citation window available to recent publications. Figure 2 complements the descriptive impact indicator reported in Table 1 by showing how citation impact varies across publication cohorts, while the temporal evolution of scientific production is examined more directly through the life-cycle analysis presented in the following section.

#### 3.1.2 Life cycle of publications

To assess the developmental trajectory and maturity of research on emotion regulation in adolescence, a life cycle analysis of scientific production was conducted using a logistic growth model. In the present dataset, the model estimates a saturation level (K) of approximately 14,485 cumulative publications, indicating the potential upper boundary of the literature on emotion regulation in adolescence. The peak year of scientific production is estimated around 2023, with an annual peak of approximately 1,078 publications, while the estimated growth duration is about 14.8 years. The goodness-of-fit indicators suggest that the model adequately captures the observed trend in the data (R^2^ = 0.830).

According to the model, the field has currently reached approximately 75% of its estimated saturation level, suggesting that research on emotion regulation in adolescence is entering a maturity phase. In this stage, scientific production continues to grow but at a progressively slower rate as the field approaches its asymptotic limit.

The milestone analysis further supports this interpretation. The model estimates that 10% of the saturation level was reached around 2016, marking the beginning of the phase of rapid expansion, while the midpoint of the growth curve (50% of K) occurred around 2023, corresponding to the period of maximum acceleration in publication output. Projections indicate that 90% of the estimated saturation level may be reached around 2030, with near-complete stabilization (approximately 99% of K) expected around 2039.

These results, illustrated in Figure 3, indicate that the literature on emotion regulation in adolescence has experienced a period of rapid expansion over the past decade and is now transitioning toward a more mature stage of development. In particular, the logistic model identifies a peak of annual scientific production around 2023, suggesting that the field has recently passed its phase of maximum growth acceleration. Although research output is expected to continue increasing in the coming years, the growth rate will likely gradually slow as the field approaches its estimated saturation level.

**Figure 3:**
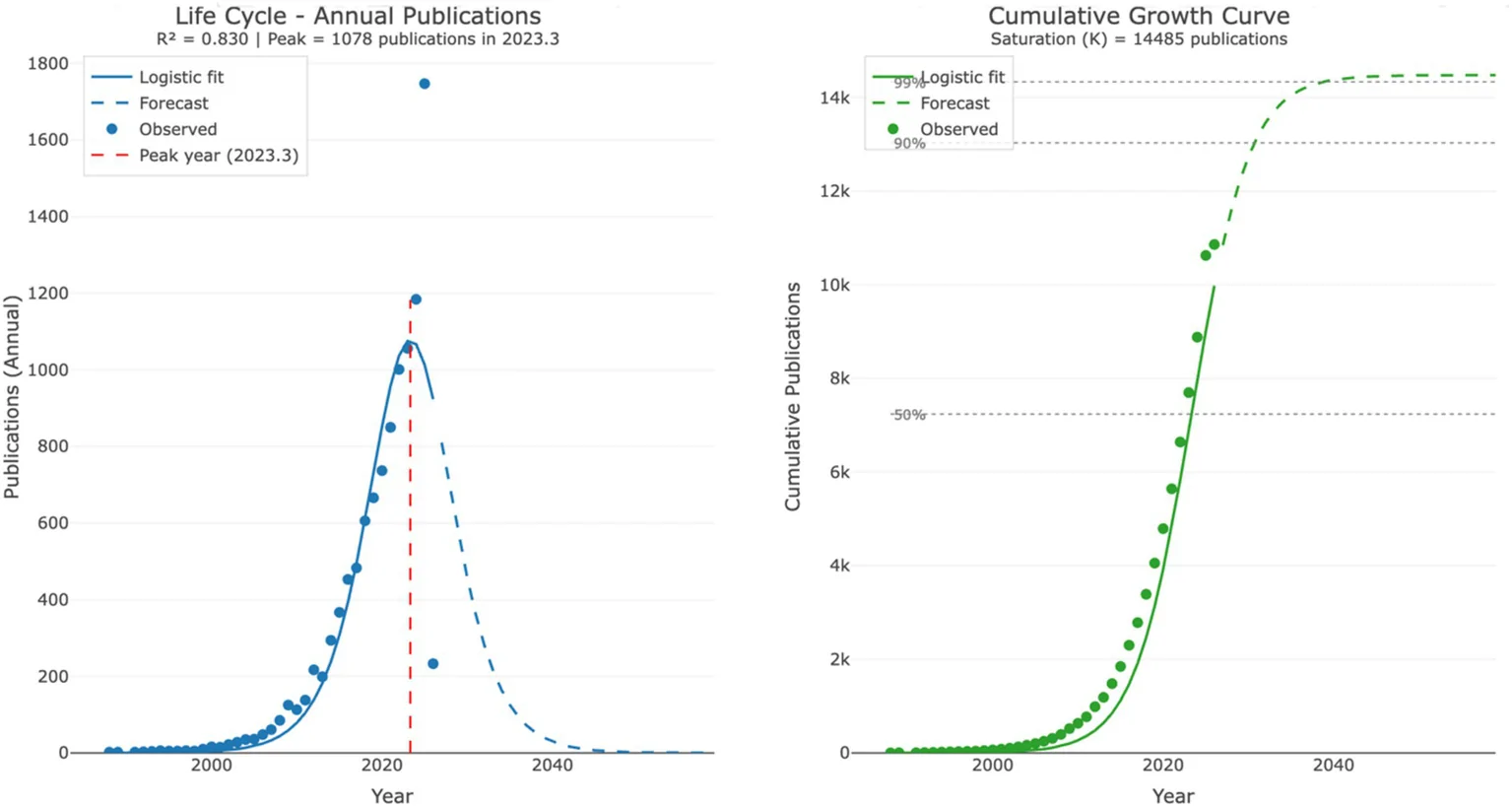
Life cycle analysis of scientific production on emotion regulation in adolescence based on a logistic growth model. The left panel shows the annual number of publications from 1988 to 2026 (observed data) together with the fitted logistic growth curve and the projected future trend, highlighting the estimated peak of annual production around 2023. The right panel presents the cumulative growth curve of publications and the corresponding logistic fit, indicating the estimated saturation level of the field (K ≈ 14,485 publications) and the progression of the research domain toward its maturity stage.

#### 3.1.3 Sources

This section examines both the most relevant sources, which capture the journals publishing the largest number of documents in the dataset, and the most locally cited sources, which identify the journals most frequently cited by the articles included in the collection (Aria and Cuccurullo, 2017).

With regard to publication productivity, the results indicate that the literature on emotion regulation in adolescence is currently distributed across a broad set of journals rather than concentrated in a few specialized outlets (Table 2). The most productive source is *Frontiers in Psychology* (261 articles), followed by *Frontiers in Psychiatry* (215) and the *Journal of Affective Disorders* (196). Other relevant publication venues include *Current Psychology* and *Development and Psychopathology*, together with several journals focused on developmental and adolescent psychology such as the *Journal of Youth and Adolescence* and the *Journal of Adolescence*. This pattern suggests that research on emotion regulation in adolescence is strongly interdisciplinary, spanning developmental psychology, clinical psychology, psychiatry, and public health.

**Table 2:** Most relevant sources.

Sources	Articles
Frontiers In Psychology	261
Frontiers In Psychiatry	215
Journal Of Affective Disorders	196
Current Psychology	171
Development And Psychopathology	165
Journal Of Child And Family Studies	139
Journal Of Youth And Adolescence	124
Journal Of Adolescence	119
Child Psychiatry & Human Development	107
Child Abuse & Neglect	99

A partially different picture emerges when examining the most locally cited sources, which highlight the intellectual structure of the field (Aria and Cuccurullo, 2017), (Table 3). The journals receiving the highest number of citations within the dataset are *Child Development*, the *Journal of the American Academy of Child and Adolescent Psychiatry*, and the *Journal of Child Psychology and Psychiatry*, followed by *Development and Psychopathology*, the *Journal of Personality and Social Psychology*, and *Developmental Psychology*. These journals represent some of the most established outlets in developmental and clinical psychology and have historically played a central role in shaping the theoretical and empirical foundations of research on emotional development and regulation.

**Table 3:** Most cited sources.

Sources	Articles
Child Development	10,954
Journal Of The American Academy Of Child And Adolescent Psychiatry	10,685
Journal Of Child Psychology And Psychiatry	9,324
Development And Psychopathology	8,867
Journal Of Personality And Social Psychology	8,189
Developmental Psychology	7,453
Journal Of Affective Disorders	7,400
Journal Of Abnormal Child Psychology	7,102
Clinical Psychology Review	7,001

This contrast between the two rankings reflects a common pattern in bibliometric studies. While the most cited journals tend to be long-standing and highly influential sources that have contributed to the conceptual and theoretical foundations of the field, the most productive journals are often more recent or multidisciplinary publication venues that currently host a growing share of empirical studies (Aria et al., 2020).

These results suggest that the literature on emotion regulation in adolescence is organized around two complementary sets of journals. On the one hand, long-established developmental and clinical psychology journals continue to provide the theoretical and conceptual foundations of the field. On the other hand, a growing number of multidisciplinary and open-access journals have increasingly become important outlets for the publication of recent empirical research.

#### 3.1.4 Most local cited documents

To identify the intellectual foundations and the specific knowledge base of the literature on emotion regulation in adolescence, an analysis of the most locally cited documents was conducted. Table 4 reports the most influential documents according to their Local Citation Score, together with their Global Citation Score and the LCS/GCS ratio. This comparison allows distinguishing between publications that are highly influential within the specific research community and those that have achieved broader recognition across multiple fields.

**Table 4:** Top 10 – most cited documents based on number of local citations.

Paper	Year	Local citations	Global citations	LC/GC ratio (%)
Morris As, 2007, Soc Dev	2007	577	2,108	27.37
Silk Js, 2003, Child Dev	2003	432	854	50.59
Zimmermann P, 2014, Int J Behav Dev	2014	398	729	54.60
Schäfer Jö, 2017, J Youth Adolescence	2017	344	695	49.50
Compas Be, 2017, Psychol Bull	2017	340	1,006	33.80
Gullone E, 2012, Psychol Assessment	2012	321	471	68.15
Mclaughlin Ka, 2011, Behav Res Ther	2011	301	553	54.43
Mcrae K, 2012, Soc Cogn Affect Neur	2012	280	530	52.83
Zeman J, 2006, J Dev Behav Pediatr	2006	266	578	46.02
Klonsky Ed, 2007, Clin Psychol Rev	2007	258	1,361	18.96

The most locally cited document is Morris et al. (2007), published in *Social Development*, which receives 577 local citations and more than 2,100 global citations. This influential paper proposes an integrative framework explaining how family context, parental socialization, and environmental factors contribute to the development of emotion regulation processes in children and adolescents (Morris et al., 2007). Notably, the single most locally cited document in the corpus is not centered on an individual regulatory strategy but on the family as the primary context in which regulation develops; an early signal that the empirical foundation of the field is relational rather than purely intrapersonal.

Another highly influential contribution is Silk et al. (2003) in *Child Development*, which examines emotion regulation processes during adolescence with a focus on emotional reactivity and interpersonal functioning. Together with Morris et al. (2007), this contribution situates the daily experience of regulation within interpersonal and family contexts, reinforcing the relational character of the field’s empirical core.

Several other highly cited papers focus on the relationship between emotion regulation and psychological adjustment. For instance, Zimmermann and Iwanski (2014) explore developmental trajectories of emotional regulation and their association with psychosocial outcomes, while Schäfer et al. (2017) investigate emotion regulation strategies among adolescents and their relationship with mental health and well-being. Similarly, McLaughlin et al. (2011) examine transdiagnostic mechanisms linking emotion regulation difficulties with psychopathological symptoms, providing empirical evidence on the role of regulatory processes in psychological disorders.

The list of highly cited documents also includes influential review and conceptual contributions. Notably, Compas et al. (2017) provides a comprehensive review of coping and emotion regulation processes across development, integrating theoretical models and empirical findings into a coherent framework. Likewise, Klonsky (2007) offers a conceptual analysis of emotion regulation functions in relation to maladaptive behaviors, particularly self-injury.

Other contributions highlight the measurement and assessment of emotion regulation. Gullone and Taffe (2012) provide important methodological insights into the assessment of emotion regulation in youth, while Zeman et al. (2006) contribute to the conceptualization and measurement of emotional competence during development. In addition, McRae et al. (2012) provide neuroscientific evidence on the neural mechanisms underlying cognitive reappraisal, linking developmental research on emotion regulation with findings from affective neuroscience.

The comparison between local and global citation scores further reveals important differences in the type of influence exerted by these studies. Some publications, such as Morris et al. (2007) and Compas et al. (2017), display both high local and global citation counts, indicating that they represent widely recognized milestones both within the specific literature on adolescent emotion regulation and within the broader psychological science community. Conversely, other works show a relatively high LCS/GCS ratio, such as Gullone and Taffe (2012) or Zimmermann and Iwanski (2014), suggesting that their influence is particularly strong within the specialized research community focusing on emotion regulation in adolescence.

#### 3.1.5 Most local cited references

To identify the intellectual foundations of the research domain, an analysis of the most cited references was conducted. Unlike the previous section, which focused on documents contained within the dataset, this analysis examines the references cited by the 10,865 publications included in the collection. In this way, it becomes possible to identify the works that form the underlying knowledge base of the field. Table 5 reports the most frequently cited references together with the number of times each work appears across the reference lists of the dataset. These publications represent the conceptual, theoretical, and methodological foundations upon which the literature on emotion regulation in adolescence has developed.

**Table 5:** Top 10-most cited reference based on number of local citations.

Cited references	Citations
Gratz Kl, 2004, J Psychopathol Behav, V26, P41	1,222
Gross Jj, 2003, J Pers Soc Psychol, V85, P348	1,048
Aldao A, 2010, Clin Psychol Rev., V30, P217	875
Gross J. J., 1998, Review Of General Psychology	728
Hu Lt, 1999, Struct Equ Modeling, V6, P1	655
Thompson R A, 1994, Monogr Soc Res Child Dev, V59, P25	601
Morris As, 2007, Soc Dev, V16, P361	577
Anonymous, 1980, Diagnostic And Statistical Manual Of Mental Disorders, V3rd	567
Anonymous, 2000, Diagnostic And Statistical Manual Of Mental Disorders: Dsm-5, V4th	551
Cohen J, 1988, Stat Power Anal Beha	523

Among the most cited works, several key contributions are directly related to the conceptualization and measurement of emotion regulation. The most frequently cited reference is Gratz and Roemer (2004), which introduced the Difficulties in Emotion Regulation Scale (DERS), one of the most widely used instruments for assessing emotion regulation difficulties.

Another foundational contribution is the work of Gross and John (2003), which examines individual differences in two major emotion regulation strategies: cognitive reappraisal and expressive suppression, and their implications for psychological functioning. This paper builds on Gross’s earlier theoretical framework (Gross, 1998), which proposed the process model of emotion regulation, a framework that has shaped the study of regulatory processes across developmental and clinical psychology.

The presence of Aldao et al. (2010) among the most cited references further highlights the relationship between emotion regulation strategies and psychopathology. This meta-analytic review systematically examined the associations between different regulation strategies and mental health outcomes, providing empirical support for the role of maladaptive regulation patterns in psychological disorders.

Several other highly cited works provide broader theoretical or developmental perspectives. For instance, Thompson (1994) offered one of the earliest definitions of emotion regulation within developmental psychology, emphasizing the processes through which individuals monitor, evaluate, and modify emotional reactions. Similarly, the prominence of Morris et al. (2007) among the top-cited references underscores the foundational role of the family context within the domain’s knowledge base. This finding is consistent with its strategic position as an intellectual bridge connecting developmental research with the broader architecture of the field.

Beyond the core conceptual literature on emotion regulation, the list of cited references also reveals the methodological foundations of the field. For example, Hu and Bentler (1999) provide guidelines for evaluating model fit in structural equation modeling, a statistical approach frequently employed in psychological research. Likewise, Cohen (1988) remains a standard reference for statistical power analysis in behavioral sciences.

Finally, the presence of the Diagnostic and Statistical Manual of Mental Disorders (DSM) among the most cited references indicates the strong connection between research on emotion regulation and clinical psychology and psychiatry.

### 3.2 Conceptual structure: thematic evolution

To explore how the conceptual structure of research on emotion regulation in adolescence has evolved over time, a thematic analysis based on author keywords was conducted. This analysis integrates three complementary perspectives: the temporal evolution of research topics, the overall lexical structure of the field, and the strategic positioning of thematic clusters. Specifically, the analysis includes (1) a word cloud visualization of the most frequent keywords, (2) trend topics analysis, and (3) a thematic map identifying the structural organization of research themes within the field.

#### 3.2.1 Word cloud

To provide a general overview of the conceptual landscape, a word cloud based on the 50 most frequent author keywords was generated (Figure 4). To reduce the tautological prominence of the most generic search-defining terms, the core keywords directly reflecting the main construct and target population were excluded from the visualization. In this representation, the size of each term corresponds to its frequency within the dataset of 10,865 publications.

**Figure 4:**
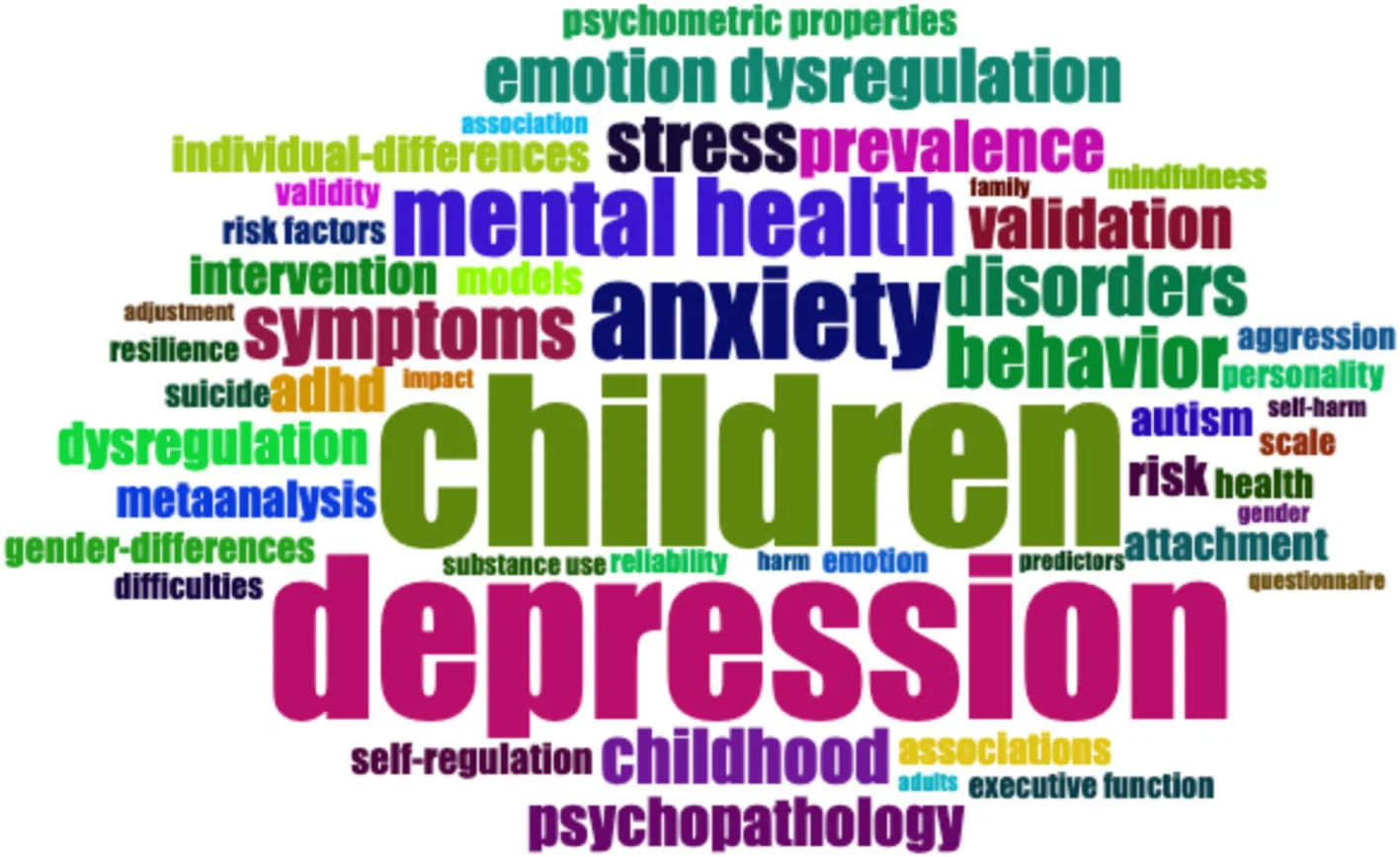
Word cloud of the 50 most frequent author keywords after exclusion of the most generic search-defining terms.

Among the remaining keywords, depression and children emerge as the most prominent terms, followed by anxiety, mental health, stress, symptoms, disorders, validation, prevalence, and emotion dysregulation. This lexical profile highlights the strong clinical orientation of the literature, with particular emphasis on internalizing symptoms, broader mental-health outcomes, and maladaptive regulatory functioning.

At the same time, the prominence of children and childhood suggests that a substantial portion of the literature situates adolescent emotion regulation within a broader developmental framework, often considering continuity across developmental stages. Additional frequently occurring terms such as attachment, individual differences, intervention, psychopathology, risk, and behavior further indicate the relevance of relational, developmental, and risk-related perspectives within the field.

Several other keywords point to more specific thematic strands that enrich the conceptual structure of the literature. These include ADHD, autism, executive function, mindfulness, resilience, self-harm, and suicide, reflecting the contribution of neurodevelopmental, psychosocial, and clinically severe outcome-oriented lines of research. Moreover, the presence of terms such as validation, psychometric properties, reliability, and questionnaire suggests continued attention to the measurement and assessment of emotion-related constructs in youth populations.

Although word clouds provide a relatively simple descriptive visualization, they are useful in offering an immediate representation of the dominant terminology used across the literature and in highlighting the broader thematic contexts in which adolescent emotion regulation research is embedded.

#### 3.2.2 Trend topics

As illustrated in Figure 5, several topics have shown substantial growth in recent years. In particular, emotion regulation, adolescence, mental health, emotion dysregulation, depression, and anxiety emerge as central themes that have experienced a marked increase in research attention since approximately 2018–2020. Their growing prominence reflects the consolidation of emotion regulation as a core construct within adolescent developmental and clinical research.

**Figure 5:**
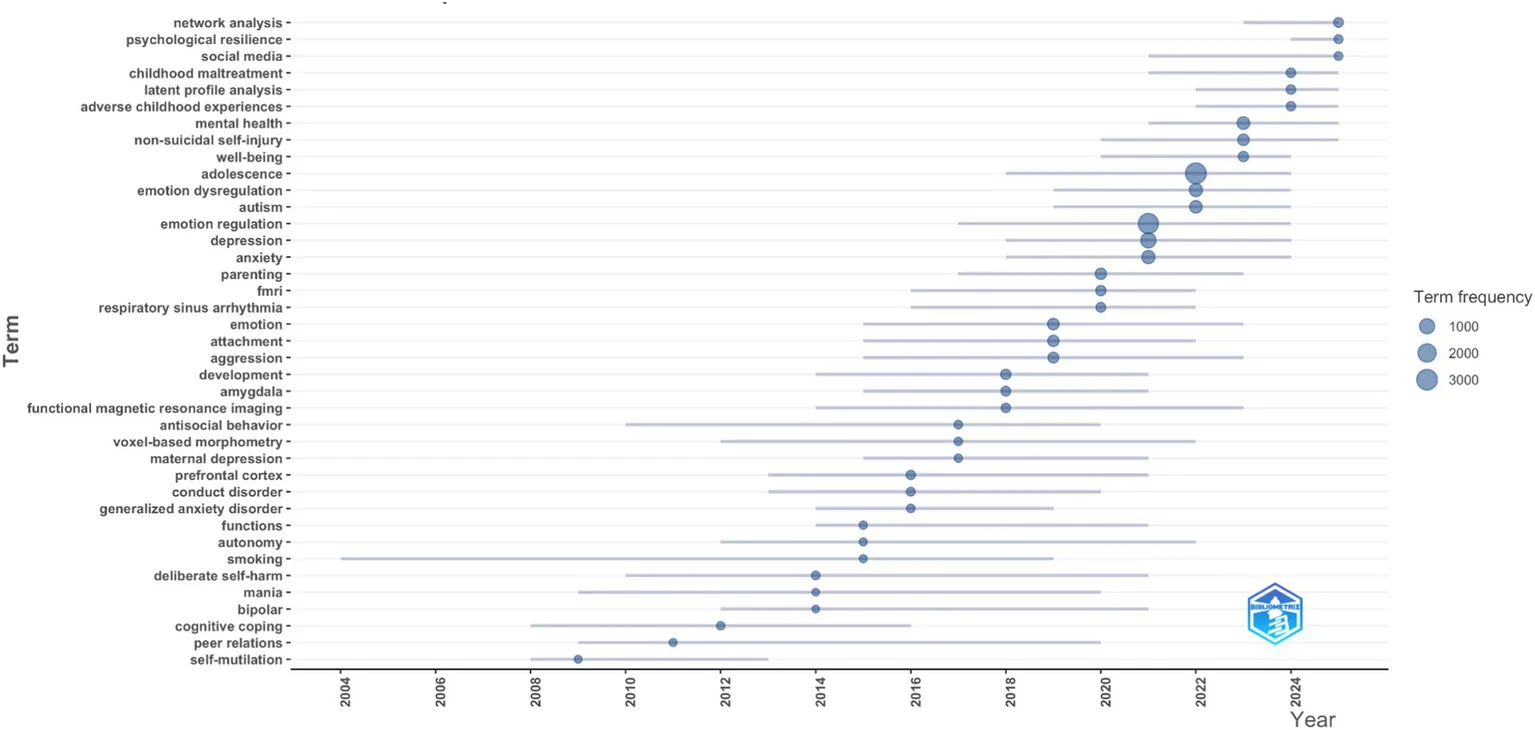
Representation of trend topic keywords over the years. The dimensions of the circles refer to the frequency of the keywords.

At the same time, the figure highlights the emergence of several methodological and contextual research directions in more recent years. Keywords such as network analysis and latent profile analysis suggest an increasing use of advanced quantitative approaches to investigate complex patterns of emotional and psychological functioning. Additionally, themes such as social media, psychological resilience, childhood maltreatment, adverse childhood experiences, and well-being appear primarily in the most recent years of the dataset, indicating growing attention to contemporary psychosocial contexts, adversity, and adaptive functioning.

Other themes show a longer historical trajectory within the literature. Topics such as attachment, parenting, aggression, respiratory sinus arrhythmia, functional magnetic resonance imaging, amygdala, and prefrontal cortex have been present across earlier phases of the field, indicating the long-standing contribution of developmental, relational, psychophysiological, and neurobiological perspectives to the study of adolescent emotion regulation.

Finally, some keywords reflect specific clinical and developmental research strands that characterized earlier phases of the literature. Terms such as self-mutilation, deliberate self-harm, bipolar disorder, mania, cognitive coping, and peer relations appear predominantly in the earlier portion of the timeline, suggesting that the field has progressively expanded from more circumscribed clinical and coping-related topics toward a broader and more diversified research landscape.

#### 3.2.3 Thematic map

As shown in Figure 6, the thematic map identifies several clusters distributed across the four quadrants of the diagram.

**Figure 6:**
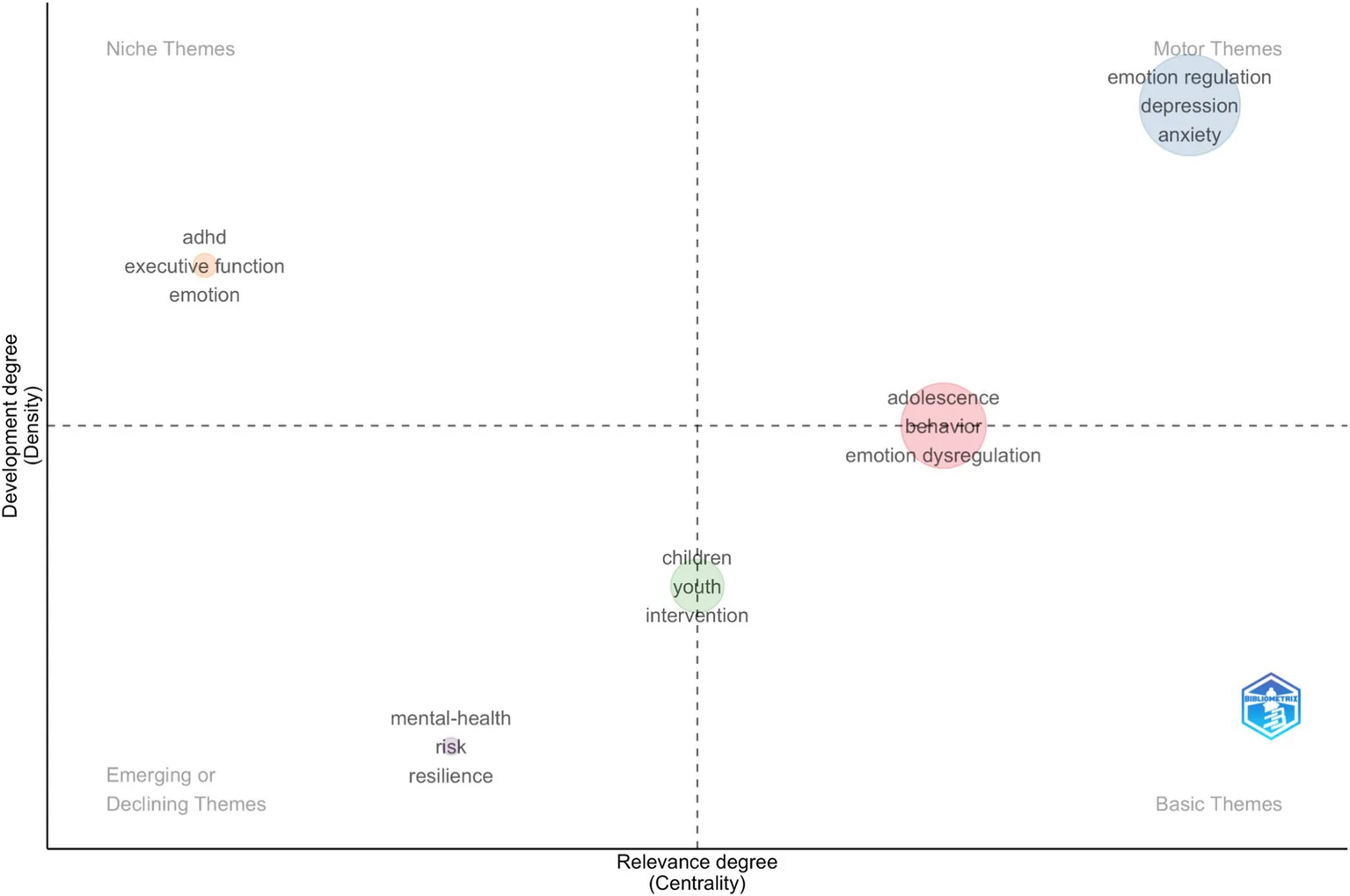
Thematic map.

In the Motor Themes quadrant (high centrality and high density), the cluster composed of emotion regulation, depression, and anxiety emerges as the core driving theme of the field. The position of this cluster indicates that research on emotion regulation is strongly integrated with the study of internalizing psychopathology, highlighting the central role of clinical and transdiagnostic perspectives within the contemporary conceptual structure of the domain.

A second cluster, composed of adolescence, behavior, and emotion dysregulation, is positioned close to the boundary between the Motor and Basic Themes quadrants. Its relatively high centrality indicates that these topics are strongly connected to the broader research field, while their intermediate density suggests a somewhat less consolidated internal development compared with the main motor theme.

In the Niche Themes quadrant (high density but lower centrality), the cluster including ADHD, executive function, and emotion identifies a more specialized research area. These themes appear to be well developed internally but comparatively less connected to the overall conceptual structure, reflecting a distinct neurodevelopmental and executive-function-oriented line of research.

Finally, the lower portion of the map includes two less developed thematic areas. The cluster composed of children, youth, and intervention lies close to the centrality threshold, suggesting a broadly relevant but less internally consolidated research stream. The cluster including mental health, risk, and resilience is located within the Emerging or Declining Themes quadrant, indicating topics that are comparatively less developed and less integrated within the overall thematic structure of the field.

### 3.3 Social structure: collaboration patterns

To examine the social structure underlying research on emotion regulation in adolescence, a collaboration analysis was conducted at two levels: authors and institutions. Collaboration networks provide insights into how scientific knowledge is produced and disseminated through partnerships among researchers and research organizations. By mapping co-authorship relationships, it is possible to identify stable research groups, influential scholars, and the institutional hubs that facilitate international collaboration within the field.

#### 3.3.1 Authors collaboration network

As shown in Figure 7, the collaboration network reveals several well-defined clusters corresponding to established research communities within the field. Among the most prominent groups is the cluster centered around Jessica Silk, which also includes researchers such as Dahl, Forbes, and Ladouceur. This cluster appears to represent a highly interconnected research group focusing primarily on adolescent emotional development and affective processes.

**Figure 7:**
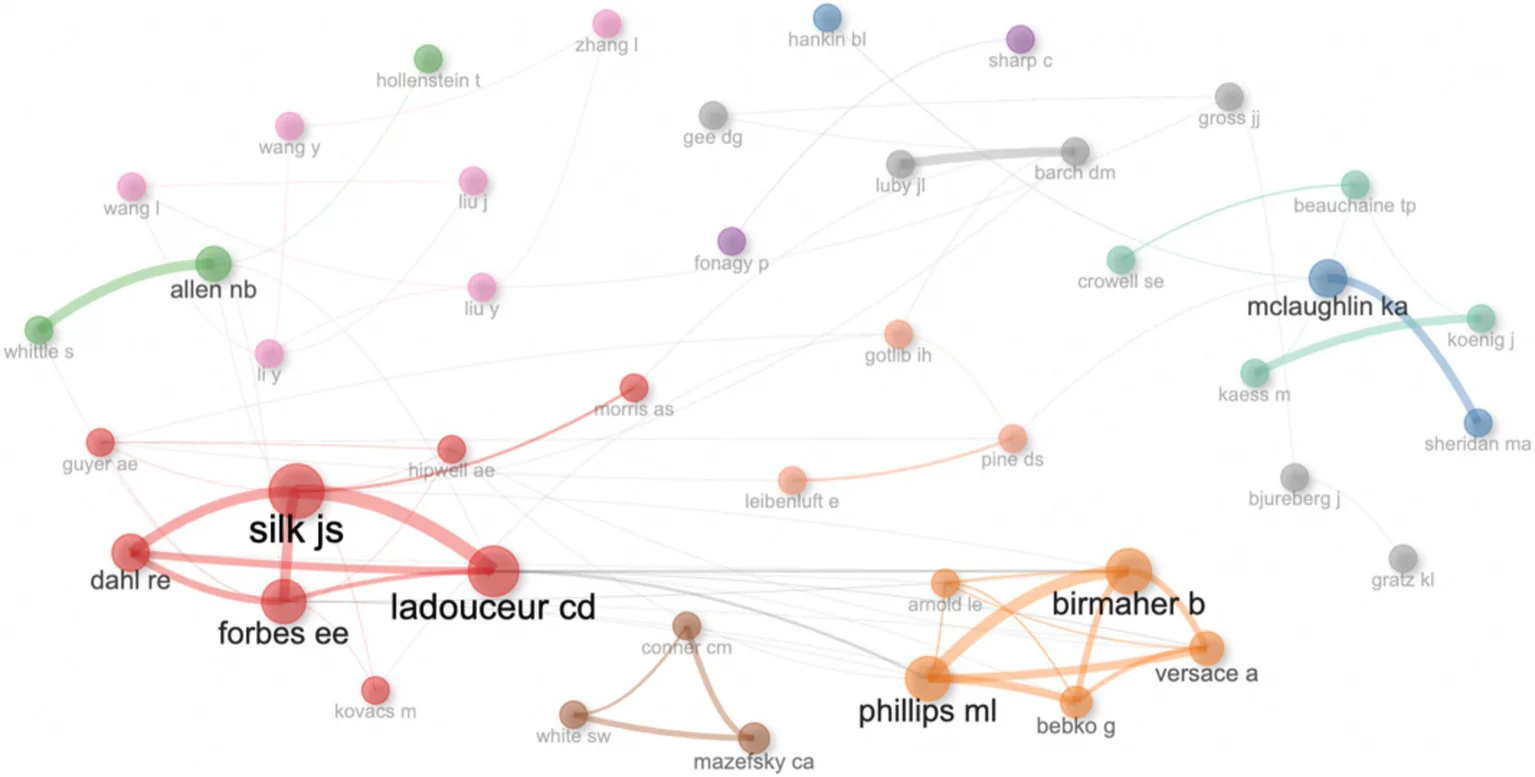
Authors’ collaboration network.

Another relevant cluster is organized around Birmaher and Phillips, which includes collaborators such as Bebko and Versace. This group appears to be strongly oriented toward research on adolescent psychopathology and mental health outcomes, reflecting the close relationship between emotion regulation and clinical psychology.

A further identifiable cluster includes authors such as McLaughlin, Sheridan, and Koenig, whose work is widely associated with research on stress exposure, adversity, and emotional dysregulation in youth. The strong internal connections within this cluster indicate stable collaboration patterns across multiple publications.

Other smaller but clearly defined communities are also visible in the network. For instance, the cluster centered on Allen and Whittle appears to represent a research group focusing on developmental and neurobiological aspects of emotion regulation during adolescence.

Overall, the network structure suggests that the field is organized around several cohesive research groups led by highly productive scholars, rather than a single dominant collaborative structure. At the same time, a number of cross-cluster connections are visible, indicating the presence of bridging authors who contribute to linking different research communities. These bridging figures play an important role in facilitating knowledge exchange between different theoretical and methodological traditions.

The network also highlights the presence of some relatively isolated authors, suggesting that certain researchers contribute to the field without being embedded in large collaborative groups. This pattern is not uncommon in interdisciplinary areas where scholars from related domains occasionally contribute to the literature.

#### 3.3.2 Institutions collaboration network

As illustrated in Figure 8, the institutional collaboration network is characterized by a highly interconnected structure dominated by several major academic hubs. Among these, the University of California system emerges as one of the most central nodes in the network, displaying numerous connections with other leading research institutions. This centrality suggests that universities within the University of California system play a pivotal role in facilitating collaborative research in the field.

**Figure 8:**
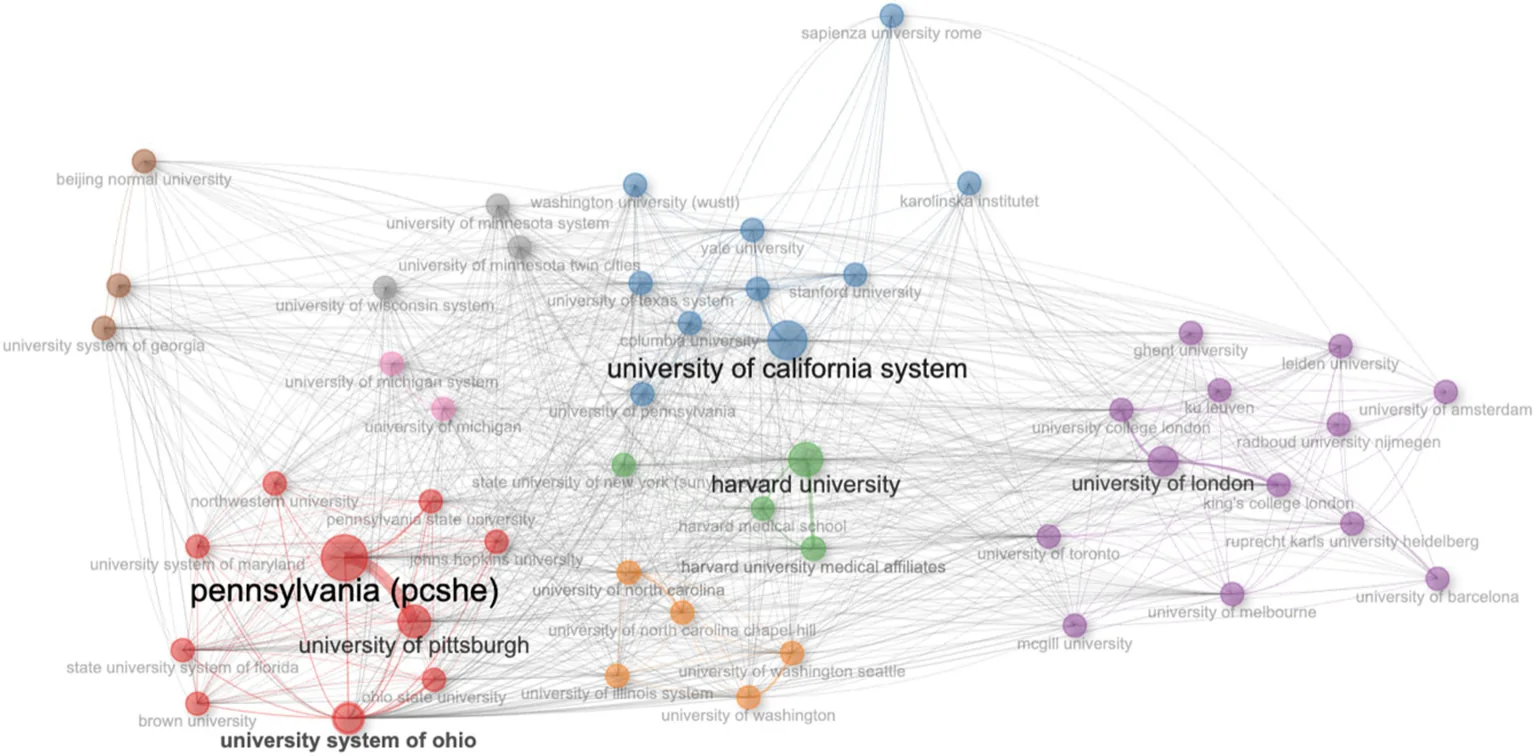
Institutions’ collaboration network.

Another highly prominent institution is Harvard University, which appears as a central hub connecting multiple clusters of institutions. The strong collaborative links between Harvard and other universities highlight its importance as a major contributor to research on adolescent emotion regulation and mental health.

The network also reveals several regional clusters reflecting patterns of geographical collaboration. For example, a large European cluster includes institutions such as University College London, King’s College London, Leiden University, KU Leuven, and Radboud University Nijmegen, indicating strong collaborative ties within the European research community.

Similarly, a cluster of North American institutions can be observed around universities such as Pennsylvania State University, University of Pittsburgh, and Ohio State University, suggesting a strong regional collaboration structure within the United States.

In addition to these major hubs, several other institutions, including Karolinska Institutet, Yale University, Stanford University, and Sapienza University of Rome, occupy strategic positions within the network and contribute to connecting different clusters. These institutions likely play an important role as bridging organizations, facilitating international collaborations and interdisciplinary exchanges.

### 3.4 Intellectual structure: foundational knowledge

Through the diachronic integration of the three temporal intervals (TF1, TF2, and TF3, Figure 9), the analysis made it possible to observe the progressive overcoming of the fragmentation of a field that, since its origins, has been described as a “topic in search of definition” (Thompson, 1994). The mapping outlines the transition from classical developmental models to contemporary clinical and transdiagnostic perspectives, identifying the theoretical anchors and intellectual turning points that have marked the consolidation of foundational knowledge in this scientific domain. To make the structural role of the most influential references directly accessible in the main text, betweenness and closeness centrality values for key works are reported within the interpretation of each temporal network. Complete centrality profiles, including strength, betweenness, and closeness for all nodes, are provided in Supplementary Table S1.

**Figure 9:**
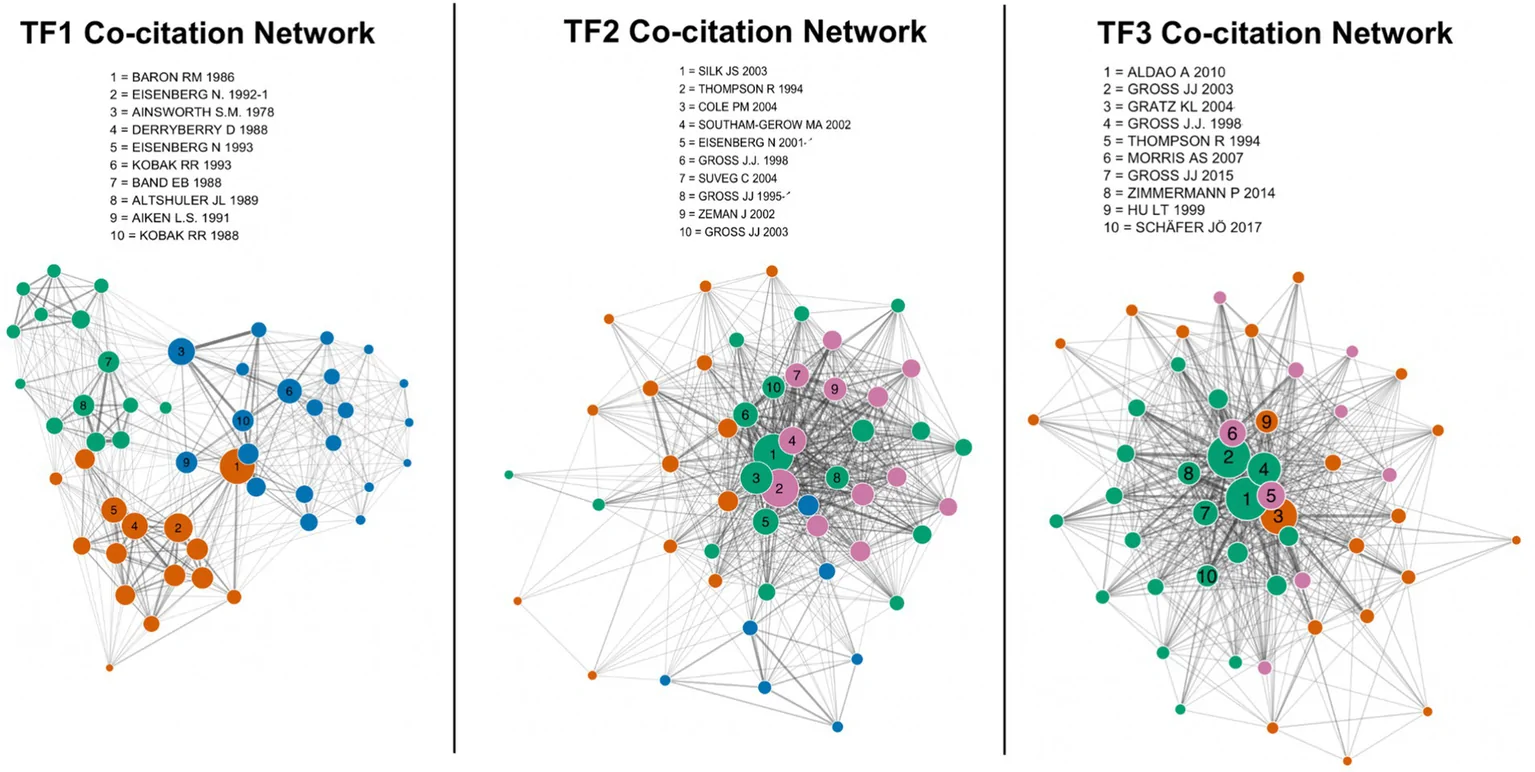
Diachronic evolution of the co-citation network of documents across three-time intervals: TF1 (1988–2000), TF2 (2001–2013) and TF3 (2014–Present).

#### 3.4.1 Time frame 1 (1988–2000): developmental foundations

In this initial phase, the intellectual structure of emotion regulation (ER) is organized around three core theoretical pillars that explain how individuals manage emotional arousal and stress.

*Orange cluster: methodology and temperament*. This cluster addresses the mechanisms through which individual traits shape behavior. Baron and Kenny (1986) represent the core of the network (betweenness = 311.190; closeness = 0.022), providing the framework for distinguishing mediators from moderators in causal models of ER. Eisenberg et al. (1992) also show substantial centrality (betweenness = 116.629; closeness = 0.020), alongside contributions from Eisenberg et al. (1993) and Derryberry and Rothbart (1988), integrating ER within temperament theory and identifying attentional processes and inhibitory control as key mechanisms regulating arousal and biological reactivity.

*Blue cluster: attachment theory and relational functioning*. Centered on the contributions of Ainsworth et al. (1978), Kobak et al. (1993), and Kobak and Sceery (1988), this group establishes the relational origins of emotion regulation. Ainsworth et al. (1978) show particularly high betweenness within TF1 (betweenness = 144.468; closeness = 0.019), indicating that attachment theory already occupied an important bridging position within the early intellectual structure. Secure attachment is conceptualized as the “secure base” necessary for the development of effective self-regulatory capacities, a perspective that remains a fundamental theoretical substrate in subsequent periods.

*Green cluster: coping strategies and stress responses*. Represented by Altshuler and Ruble (1989) and Band and Weisz (1988), this cluster focuses on conscious efforts to manage environmental demands. These studies highlight the shift from primary control (acting on the environment) to secondary control (acting on the self), emphasizing behavioral and cognitive distraction as key regulatory strategies in the face of uncontrollable stress.

#### 3.4.2 Time frame 2 (2001–2013): models’ formalization and clinical expansion

In this phase, the intellectual structure expands and becomes more integrated, organizing into four distinct thematic clusters.

*Pink cluster: the process model and everyday life*. This cluster represents the core of the network in this period. Silk et al. (2003) occupy the most structurally central position in TF2, with the highest betweenness and closeness values observed in this temporal network (betweenness = 508.000; closeness = 0.180). Their work on emotional variability in everyday life therefore functions not only as an influential empirical contribution but also as a major connective node within the emerging intellectual structure. Alongside them, the works of Gross (1998), Gross and John (2003), and Cole et al. (2004) formalize the process model of emotion regulation, distinguishing between antecedent-focused strategies and response-focused strategies.

*Green cluster: from development to psychopathology*. Anchored by Thompson (1994), this cluster acts as a bridge between developmental and clinical psychology. This structural role is also reflected in Thompson’s high centrality within TF2 (betweenness = 303.000; closeness = 0.174), second only to Silk et al. (2003) in betweenness centrality. Together with Southam-Gerow and Kendall (2002), Suveg and Zeman (2004), and Zeman et al. (2002), this cluster shifts the focus toward emotional understanding and the ways in which regulatory deficits manifest in anxiety disorders and internalizing symptoms during childhood and adolescence.

*Orange cluster: clinical dysregulation and measurement*. This cluster retains the methodological legacy of Baron and Kenny (1986), while integrating the contribution of Gratz and Roemer (2004) on the DERS scale. This inclusion shifts the focus toward the clinical multidimensionality of dysregulation, encompassing deficits in awareness, clarity, and impulse control.

*Blue cluster: the roots of attachment*. This cluster continues to represent the relational foundations of regulation through Cassidy (1994), Kobak et al. (1993), and Kobak and Sceery (1988). Kobak et al. (1993) retain a meaningful bridging role within TF2 (betweenness = 87.000; closeness = 0.091), supporting the persistence of attachment-related perspectives within the increasingly integrated intellectual structure.

#### 3.4.3 Time frame 3 (2014-present): clinical integration and global vision

In this final phase, the intellectual structure reaches its highest density, reflecting an increasingly integrated ecosystem where developmental and clinical boundaries merge into a transdiagnostic and methodologically rigorous framework.

*Green cluster: transdiagnostic synthesis*. As the gravitational center, this cluster is led by Aldao et al. (2010), whose meta-analysis formalizes the transdiagnostic perspective by identifying specific strategies such as rumination and suppression as common risk factors across disorders. Aldao et al. (2010) show the highest betweenness centrality in TF3 (betweenness = 467.000; closeness = 1.225), indicating a particularly prominent connective position within the contemporary knowledge base. Gratz and Roemer (2004) also occupy a highly central structural position (betweenness = 440.000; closeness = 1.139), reflecting the integration of multidimensional emotion dysregulation within the contemporary network. Gross and John (2003) constitute a further major bridging reference (betweenness = 391.000; closeness = 1.169), highlighting the continued structural relevance of process-based models of emotion regulation.

*Orange cluster: family systems and foundational theory*. Maintaining continuity with TF1 and TF2, this cluster is anchored by the tripartite model of Morris et al. (2007), focusing on how family context, parental socialization, and emotional climate shape adolescents’ regulatory capacities. The persistent centrality of Thompson (1994) confirms that his original definition of ER remains the fundamental theoretical anchor, linking biology with relational dynamics.

*Pink cluster: trajectories of growth and emotional stability*. Represented by Schäfer et al. (2017) and Zimmermann and Iwanski (2014), this cluster specializes in the stabilization of ER patterns from adolescence to adulthood, examining how the frequency and quality of specific strategies become predictive of long-term well-being. It continues the developmental-dynamics line previously associated with Silk et al. (2003) in TF2.

### 3.5 Sensitivity analysis

The sensitivity analysis showed that the principal network structures were largely robust to document-type restriction. The authors’ collaboration network preserved its main nodes and collaborative communities (Supplementary Figure S3). Greater variation was observed in the thematic map, particularly in the composition and strategic positioning of some clusters (Supplementary Figure S4), although the underlying conceptual structure remained substantially preserved, as confirmed by the stability of the author-keyword co-occurrence network (Supplementary Figure S5). Finally, the reference co-citation networks retained the same leading references and overall intellectual configuration across all three temporal intervals (Supplementary Figure S6).

## 4 Discussion

The bibliometric analysis delineates a domain that has progressively moved beyond the fragmentation that historically characterized emotion regulation research. Overall, the findings portray adolescent emotion regulation as an increasingly mature and interconnected research field, structured around identifiable intellectual, conceptual, and collaborative patterns.

From a life-cycle perspective, the field appears to be approaching a phase of maturity after a prolonged period of rapid expansion. Rather than indicating a loss of scientific interest, the expected deceleration in publication growth is consistent with a process of consolidation, in which increasing attention may be directed toward refining, integrating, and differentiating established models and research lines. Thus, the developmental trajectory of the literature suggests a transition from a predominantly expansion-driven phase toward one characterized by greater theoretical and methodological organization.

The distribution of publications across scientific sources further supports this interpretation. Established developmental and clinical psychology journals remain important citation anchors (e.g., *Child Development*, *Journal of the American Academy of Child and Adolescent Psychiatry*, *Journal of Child Psychology and Psychiatry*, and *Development and Psychopathology*), reflecting the historical foundations of the field, whereas current scientific production is distributed across a broader range of multidisciplinary, clinical, and psychiatric outlets (e.g., *Frontiers in Psychology*, *Frontiers in Psychiatry*, and *Journal of Affective Disorders*). This pattern suggests not a replacement of the developmental tradition, but rather an expansion of adolescent ER research beyond its original disciplinary boundaries. The field increasingly operates at the intersection of developmental psychology, psychopathology, neuroscience, and mental health research, reflecting the progressive integration of perspectives that were historically more distinct. This pattern is broadly consistent with the bibliometric assessment of the broader emotion regulation literature by Rahaman et al. (2024), who documented substantial growth of the field and identified a heterogeneous thematic landscape encompassing clinical, developmental, relational, and neurocognitive topics. In particular, depression, anxiety, psychopathology, parenting, adolescence, and emotion dysregulation emerged prominently across their keyword and thematic analyses. The present adolescent-focused mapping refines this broader picture by showing that, within this developmental period, clinical and transdiagnostic themes occupy a particularly central position while developmental and relational perspectives remain structurally visible.

At the conceptual level, the thematic structure points to a strong contemporary convergence between emotion regulation and clinical and transdiagnostic research. The Motor Theme composed of emotion regulation, depression, and anxiety indicates that the current research front is strongly organized around the links between regulatory processes and internalizing psychopathology. At the same time, the cluster combining adolescence, behavior, and emotion dysregulation occupies a highly central position, while neurodevelopmental, developmental, and psychosocial themes remain represented in more specialized or less consolidated areas of the map. Overall, this configuration suggests that the contemporary conceptual structure integrates developmental perspectives with an increasingly prominent clinical orientation. Read across the three Time Frames, the relational foundations of regulation suggest notable continuity. The attachment cluster (Ainsworth, Kobak, Cassidy) constitutes an identifiable community in both TF1 and TF2, and in TF3 its concerns are not abandoned but absorbed into the family-systems pole anchored by Morris et al. (2007). Rather than appearing to fade as the field matures, the relational dimension appears to be carried forward, shifting from a distinct attachment community toward an integrated family- and relational-oriented cluster. This configuration also helps position the present findings in relation to previous attempts to map adolescent ER research. Using a text-mining systematic review of 6,305 publications, van Lissa (2022) described the literature as fragmented across subdisciplines and lacking an overarching theoretical framework, with relatively sparse connections among the phenomena represented in the co-occurrence networks. At the same time, that review already identified a central association between emotion regulation and mental health outcomes, alongside prominent socialization and neurocognitive themes. The present findings extend this earlier picture. Although differences in methods and temporal coverage preclude a direct comparison of network density, the current conceptual and intellectual structures suggest that the literature now displays more clearly identifiable points of integration across previously differentiated research traditions. In particular, the central clinical–transdiagnostic axis and the persistence of attachment- and family-oriented intellectual communities are consistent with a field in which developmental, relational, and psychopathological perspectives have become increasingly interconnected.

This continuity points to a broader interpretive thread running through the results. The prominence of Morris et al. (2007) and Silk et al. (2003) among the most locally cited documents, together with the persistence of attachment- and family-oriented communities across the intellectual structure, suggests that interpersonal processes constitute a durable component of the field rather than a peripheral or transient line of inquiry. Beyond the family context, peer interaction represents a further interpersonal arena of regulation, as reflected in dedicated work on peers’ responses to adolescents’ emotional expression (Delios et al., 2023). At the same time, recent trends indicate increasing methodological diversification, including the use of Network Analysis (Azevedo et al., 2025) and Latent Profile Analysis (Grommisch et al., 2020). These developments are consistent with growing interest in ER flexibility and regulatory repertoires, moving beyond the evaluation of isolated strategies toward the study of how different regulatory resources may be deployed according to contextual demands (Bonanno and Burton, 2013; Haag et al., 2024). This interpretation is consistent with the systematic review by Haag et al. (2024), which identified adolescent ER flexibility as a promising but still limited and conceptually heterogeneous research area, organized primarily around the regulation of emotional expression and the repertoire of regulatory strategies. Their findings further highlight the need to examine context sensitivity, feedback processes, and motivational components of flexible regulation. Accordingly, the prominence of flexibility-related approaches in the recent literature may be better understood as an emerging research frontier rather than an already consolidated subfield.

From a social perspective, the collaboration networks reveal a field organized around several cohesive research communities rather than a single dominant collaborative group. The coexistence of stable author clusters and cross-cluster connections suggests that knowledge production is supported both by specialized research teams and by exchanges across different theoretical and methodological traditions. At the institutional level, the network similarly points to the coexistence of strong regional research hubs and broader international connections, reflecting an increasingly interconnected scientific community.

At the same time, the prominence of North American and European institutions within the collaboration network raises questions about the geographical and cultural breadth of the research landscape. Institutional location cannot be taken as a direct indicator of the populations represented in individual studies, and the present analysis therefore does not establish that adolescent ER research is predominantly based on WEIRD (Western, Educated, Industrialized, Rich, and Democratic) samples. Nevertheless, the prominence of these regional research hubs highlights the importance of broadening future research to a wider range of sociocultural settings, including low- and middle-income countries. Greater cross-cultural attention is particularly needed to examine whether the use, effectiveness, and contextual appropriateness of regulatory strategies, as well as family- and peer-related regulatory processes, generalize across cultural settings or are shaped by locally specific norms and social environments. Such work would help clarify the extent to which current models capture broadly shared regulatory processes or culturally contingent patterns.

The diachronic co-citation analysis further suggests a progressive theoretical reorganization rather than a simple replacement of earlier perspectives. The movement from coping, temperament, and attachment-based foundations in TF1, through the formalization of process models and the increasing connection with psychopathology in TF2, to the prominence of transdiagnostic perspectives in TF3 indicates a gradual integration of previously more differentiated research traditions. Importantly, earlier developmental and relational foundations remain visible within the contemporary intellectual structure, alongside the growing prominence of clinical and transdiagnostic models. The evolution of the field therefore appears to be characterized less by successive theoretical discontinuities than by the progressive incorporation of established perspectives into a more interconnected knowledge base.

Despite the consistency of the main structural patterns identified, some considerations should be taken into account when interpreting these findings. The analysis relies exclusively on the Web of Science Core Collection, which, although widely recognized as a standard reference in bibliometric research, may not fully capture the entire body of relevant literature, particularly contributions indexed in other databases or published in non-English languages. The corpus also included heterogeneous document types; however, sensitivity analyses excluding Meeting Abstracts and Letters showed that the principal network structures were largely preserved, although the thematic map proved comparatively more sensitive to document-type restriction.

Moreover, bibliometric and network-based approaches capture patterns of scientific production and citation practices rather than direct theoretical or empirical relationships. As such, the intellectual and conceptual structures identified in this study should be interpreted as representations of the organization of the literature, rather than exhaustive reconstructions of the underlying theoretical landscape.

Finally, as with all time-dependent citation analyses, more recent developments may be only partially represented due to citation lag effects. Future research could address these aspects by integrating multiple databases and combining bibliometric techniques with complementary qualitative approaches, thereby enabling a more fine-grained understanding of the field.

## 5 Conclusion

The present work provides a comprehensive bibliometric mapping of the literature on emotion regulation in adolescence, tracing the development of the field from its early developmental and relational foundations to its contemporary clinical and transdiagnostic organization. Overall, the findings suggest that adolescent emotion regulation has become an increasingly structured and interconnected research domain, while retaining multiple theoretical traditions and areas of ongoing development.

Three main conclusions can be drawn. First, the contemporary conceptual structure is strongly organized around the intersection between emotion regulation and internalizing psychopathology, with depression and anxiety forming the principal Motor Theme. At the same time, the diachronic intellectual structure indicates that developmental and relational perspectives have remained visible throughout the evolution of the field. Against the developmental backdrop of increasing autonomy during adolescence (Ruiz and Yabut, 2024; Casey et al., 2010; Steinberg, 1990), the persistence of attachment- and family-oriented communities across the temporal intervals suggests that interpersonal contexts continue to represent an important component of the literature on adolescent emotion regulation (Ainsworth et al., 1978; Kobak et al., 1993; Morris et al., 2007).

Second, the intellectual development of the field appears to reflect a progressive theoretical reorganization rather than a sequence of discrete theoretical replacements. Early work on coping, temperament, and attachment was progressively complemented by process-oriented models and, more recently, by transdiagnostic perspectives emphasizing emotion regulation as a process relevant across multiple forms of psychopathology (Gross, 1998; Aldao et al., 2010). The resulting structure therefore suggests increasing integration between developmental, relational, and clinical approaches.

Third, the field continues to develop in new conceptual and methodological directions. Recent attention to emotion regulation flexibility and regulatory repertoires reflects growing interest in how adolescents adapt regulatory strategies to changing contextual demands, although this area remains conceptually and methodologically heterogeneous (Bonanno and Burton, 2013; Haag et al., 2024). At the same time, the emergence of approaches such as Network Analysis and Latent Profile Analysis points to increasing methodological diversification in the study of regulatory processes (Azevedo et al., 2025; Grommisch et al., 2020).

The social structure of the literature also identifies an important priority for future research. The strong representation of North American and European institutions in the collaboration network raises questions about the geographical and cultural breadth of the research landscape (Rahaman et al., 2024). Although institutional affiliation cannot be used to infer the characteristics of the samples included in individual studies, the prominence of these regional research hubs highlights the importance of broadening future research to a wider range of sociocultural settings, including low- and middle-income countries (Rahaman et al., 2024; van Lissa, 2022). Cross-cultural research will be particularly important for determining which regulatory processes and relational influences are broadly generalizable and which may depend on specific sociocultural contexts (Haag et al., 2024; van Lissa, 2022).

## Data Availability

The raw data supporting the conclusions of this article will be made available by the authors, without undue reservation.
