# Supplementary material for: From individual to relational: a scientometric mapping of emotion regulation in adolescence
Source: Front Psychol. 2026 Oct 8;17:1965549. doi: 10.3389/fpsyg.2026.1965549 (PMC13686872; doi:10.3389/fpsyg.2026.1965549)
Supplement: Supplementary file 1 [file Table_1.DOCX]

**Supplementary Materials**

**Table S1**. Centrality indices (strength, betweenness, and closeness) for the top cited references across the three temporal intervals (TF1, TF2, TF3) in the reference co-citation networks. References are grouped by timeframe and cluster membership.

| **timeframe** | **reference** | **cluster** | **strength** | **betweenness** | **closeness** |
| --- | --- | --- | --- | --- | --- |
| TF1 | BARON RM 1986 | 1 | 49 | 311.19 | 0.022 |
| TF1 | EISENBERG N. 1992 | 1 | 41 | 116.629 | 0.02 |
| TF1 | HOFFMAN M.L. 1982 | 1 | 30 | 61.32 | 0.017 |
| TF1 | BOLLEN K.A. 1989 | 1 | 23 | 30.625 | 0.018 |
| TF1 | DERRYBERRY D 1988 | 1 | 38 | 18.846 | 0.018 |
| TF1 | ROTHBART MK 1994 | 1 | 32 | 18.369 | 0.017 |
| TF1 | EISENBERG N 1993 | 1 | 37 | 11.036 | 0.018 |
| TF1 | EISENBERG N 1994 | 1 | 21 | 2.5 | 0.014 |
| TF1 | CASPI A 1995 | 1 | 32 | 0.736 | 0.017 |
| TF1 | ROBINS RW 1996 | 1 | 32 | 0.736 | 0.017 |
| TF1 | EISENBERG N 1997 | 1 | 30 | 0.369 | 0.017 |
| TF1 | EISENBERG N 1991 | 1 | 27 | 0.279 | 0.016 |
| TF1 | WATSON D 1992 | 1 | 14 | 0.25 | 0.013 |
| TF1 | EISENBERG N 1995 | 1 | 25 | 0 | 0.017 |
| TF1 | SANSON A 1990 | 1 | 31 | 0 | 0.017 |
| TF1 | AINSWORTH S.M. 1978 | 2 | 39 | 144.468 | 0.019 |
| TF1 | KOBAK RR 1993 | 2 | 36 | 94.233 | 0.018 |
| TF1 | AIKEN L.S. 1991 | 2 | 32 | 35.296 | 0.018 |
| TF1 | DOWNEY G 1990 | 2 | 21 | 21 | 0.016 |
| TF1 | HAZAN C 1987 | 2 | 31 | 20.25 | 0.018 |
| TF1 | KOBAK RR 1988 | 2 | 32 | 14.283 | 0.019 |
| TF1 | BOWLBY J. 1980-1 | 2 | 27 | 6.319 | 0.018 |
| TF1 | BOWLBY J. 1980-2 | 2 | 27 | 6.319 | 0.018 |
| TF1 | COLLINS NL 1990 | 2 | 29 | 3.667 | 0.017 |
| TF1 | BOWLBY J. 1982-1 | 2 | 24 | 1.367 | 0.017 |
| TF1 | ANONYMOUS 1985 1985 | 2 | 26 | 0 | 0.016 |
| TF1 | CROWELL JA 1988 | 2 | 22 | 0 | 0.016 |
| TF1 | CUMMINGS E.M. 1990 | 2 | 25 | 0 | 0.015 |
| TF1 | GEORGE C 1985-2 | 2 | 25 | 0 | 0.015 |
| TF1 | SPITZER R.L. 1990 | 2 | 25 | 0 | 0.015 |
| TF1 | MARKUS HR 1991 | 2 | 17 | 0 | 0.014 |
| TF1 | PLOMIN R. 1986 | 2 | 17 | 0 | 0.014 |
| TF1 | ABRAMSON LY 1978 | 2 | 16 | 0 | 0.014 |
| TF1 | PETERSON C 1984 | 2 | 16 | 0 | 0.014 |
| TF1 | ABIDIN R.R. 1986 | 2 | 17 | 0 | 0.014 |
| TF1 | HOLLINGSHEAD AB 1975 | 2 | 15 | 0 | 0.013 |
| TF1 | ALTSHULER JL 1989 | 3 | 32 | 86.483 | 0.019 |
| TF1 | BAND EB 1988 | 3 | 32 | 51.369 | 0.017 |
| TF1 | CAMPOS JJ 1989 | 3 | 28 | 34.163 | 0.015 |
| TF1 | COMPAS BE 1987 | 3 | 24 | 23.533 | 0.018 |
| TF1 | COMPAS BE 1988 | 3 | 29 | 9.75 | 0.017 |
| TF1 | SAARNI C 1990 | 3 | 23 | 2.836 | 0.015 |
| TF1 | LAZARUS R.S. 1991 | 3 | 27 | 1.75 | 0.016 |
| TF1 | MILLER S.M. 1985 | 3 | 26 | 0.5 | 0.016 |
| TF1 | BARRETT K.C. 1987 | 3 | 22 | 0.245 | 0.014 |
| TF1 | FUCHS D 1988 | 3 | 22 | 0.245 | 0.014 |
| TF1 | SAARNI C 1979 | 3 | 22 | 0.245 | 0.014 |
| TF1 | SAARNI C. 1989 | 3 | 22 | 0.245 | 0.014 |
| TF1 | MISCHEL HN 1983 | 3 | 18 | 0 | 0.015 |
| TF1 | COLLINS W.A. 1990 | 3 | 20 | 0 | 0.014 |
| TF2 | SILK JS 2003 | 1 | 579 | 508 | 0.18 |
| TF2 | COLE PM 2004 | 1 | 444 | 56 | 0.155 |
| TF2 | EISENBERG N 2001 | 1 | 344 | 40.5 | 0.144 |
| TF2 | EISENBERG N 2000 | 1 | 199 | 3.5 | 0.107 |
| TF2 | MORRIS AS 2007 | 1 | 258 | 3 | 0.13 |
| TF2 | SPEAR LP 2000 | 1 | 121 | 3 | 0.103 |
| TF2 | GROSS JJ 2003 | 1 | 290 | 2 | 0.13 |
| TF2 | GROSS J.J. 1998 | 1 | 326 | 1 | 0.142 |
| TF2 | EISENBERG N 1998 | 1 | 248 | 0.5 | 0.115 |
| TF2 | GROSS JJ 1995-1 | 1 | 296 | 0 | 0.133 |
| TF2 | GARBER J 1995 | 1 | 283 | 0 | 0.133 |
| TF2 | CAMPOS JJ 2004 | 1 | 223 | 0 | 0.12 |
| TF2 | SHEEBER L 2000 | 1 | 197 | 0 | 0.119 |
| TF2 | EISENBERG N 1996 | 1 | 213 | 0 | 0.118 |
| TF2 | HANKIN BL 1998 | 1 | 169 | 0 | 0.116 |
| TF2 | GROSS JJ 1998 | 1 | 218 | 0 | 0.113 |
| TF2 | COMPAS BE 2001 | 1 | 163 | 0 | 0.104 |
| TF2 | ROTHBART M. 1998 | 1 | 166 | 0 | 0.101 |
| TF2 | KOPP CB 1989 | 1 | 158 | 0 | 0.09 |
| TF2 | CLARK LA 1991 | 1 | 147 | 0 | 0.087 |
| TF2 | GIEDD JN 1999 | 1 | 62 | 0 | 0.067 |
| TF2 | GRATZ KL 2004 | 2 | 232 | 67 | 0.128 |
| TF2 | BARON RM 1986 | 2 | 249 | 25.5 | 0.126 |
| TF2 | LINEHAN M.M. 1993 | 2 | 174 | 12 | 0.105 |
| TF2 | ANONYMOUS 2000 | 2 | 195 | 9.5 | 0.1 |
| TF2 | MITTAL VA 2011 | 2 | 170 | 5 | 0.108 |
| TF2 | ACHENBACH T.M. 1991 | 2 | 144 | 1.5 | 0.09 |
| TF2 | AIKEN L.S. 1991 | 2 | 141 | 0 | 0.098 |
| TF2 | KAUFMAN J 1997 | 2 | 109 | 0 | 0.085 |
| TF2 | KOVACS M 1985 | 2 | 109 | 0 | 0.079 |
| TF2 | COHEN J 1988 | 2 | 98 | 0 | 0.071 |
| TF2 | HU LT 1999 | 2 | 83 | 0 | 0.07 |
| TF2 | CRICK NR 1994 | 2 | 54 | 0 | 0.057 |
| TF2 | MOFFITT TE 1993 | 2 | 65 | 0 | 0.055 |
| TF2 | THOMPSON R 1994 | 3 | 535 | 303 | 0.174 |
| TF2 | SOUTHAM-GEROW MA 2002 | 3 | 361 | 17 | 0.142 |
| TF2 | CICCHETTI D 1995 | 3 | 268 | 1 | 0.122 |
| TF2 | SUVEG C 2004 | 3 | 311 | 0 | 0.14 |
| TF2 | ZEMAN J 2002 | 3 | 295 | 0 | 0.135 |
| TF2 | COLE P.M. 1994 | 3 | 282 | 0 | 0.131 |
| TF2 | ZEMAN J 2006 | 3 | 230 | 0 | 0.126 |
| TF2 | SHIELDS A 1997 | 3 | 235 | 0 | 0.121 |
| TF2 | ZEMAN J 2001 | 3 | 253 | 0 | 0.121 |
| TF2 | SAARNI C. 1999 | 3 | 217 | 0 | 0.113 |
| TF2 | KOBAK RR 1993 | 4 | 162 | 87 | 0.091 |
| TF2 | CASSIDY J 1994 | 4 | 189 | 13.5 | 0.111 |
| TF2 | KOBAK RR 1988 | 4 | 133 | 11 | 0.085 |
| TF2 | MAIN M 1985 | 4 | 98 | 0 | 0.075 |
| TF2 | AINSWORTH S.M. 1978 | 4 | 105 | 0 | 0.074 |
| TF2 | COOPER ML 1998 | 4 | 93 | 0 | 0.069 |
| TF3 | GRATZ KL 2004 | 1 | 2983 | 440 | 1.139 |
| TF3 | HU LT 1999 | 1 | 1656 | 78 | 0.866 |
| TF3 | ANONYMOUS 2000 | 1 | 1005 | 1 | 0.691 |
| TF3 | NEUMANN A 2010 | 1 | 946 | 0 | 0.847 |
| TF3 | WEINBERG A 2009 | 1 | 874 | 0 | 0.827 |
| TF3 | LINEHAN M.M. 1993 | 1 | 868 | 0 | 0.809 |
| TF3 | ANONYMOUS 1980 | 1 | 941 | 0 | 0.727 |
| TF3 | COHEN J 1988 | 1 | 1078 | 0 | 0.697 |
| TF3 | HAYES A 2013 | 1 | 803 | 0 | 0.608 |
| TF3 | KLONSKY ED 2007 | 1 | 383 | 0 | 0.587 |
| TF3 | MUTHEN L.K. 1998 | 1 | 842 | 0 | 0.581 |
| TF3 | PREACHER KJ 2008 | 1 | 659 | 0 | 0.574 |
| TF3 | WATSON D 1988 | 1 | 625 | 0 | 0.547 |
| TF3 | KLINE R.B. 2005 | 1 | 656 | 0 | 0.535 |
| TF3 | RADLOFF L 1977 | 1 | 553 | 0 | 0.524 |
| TF3 | KAUFMAN J 1997 | 1 | 640 | 0 | 0.523 |
| TF3 | GRATZ K.L. 2008 | 1 | 640 | 0 | 0.5 |
| TF3 | GOODMAN R 1997 | 1 | 660 | 0 | 0.493 |
| TF3 | CARTER MJ 2014 | 1 | 400 | 0 | 0.469 |
| TF3 | SHAW P 2014 | 1 | 445 | 0 | 0.425 |
| TF3 | ALDAO A 2010 | 2 | 3540 | 467 | 1.225 |
| TF3 | GROSS JJ 2003 | 2 | 3538 | 391 | 1.169 |
| TF3 | GROSS J.J. 1998 | 2 | 2668 | 0 | 1.069 |
| TF3 | GROSS JJ 2015 | 2 | 1882 | 0 | 0.938 |
| TF3 | SCHÄFER JÖ 2017 | 2 | 1572 | 0 | 0.9 |
| TF3 | ZIMMERMANN P 2014 | 2 | 1689 | 0 | 0.899 |
| TF3 | GULLONE E 2012 | 2 | 1382 | 0 | 0.885 |
| TF3 | GROSS JJ 1998 | 2 | 1227 | 0 | 0.883 |
| TF3 | JOHN OP 2004 | 2 | 1187 | 0 | 0.851 |
| TF3 | MCLAUGHLIN KA 2011 | 2 | 1419 | 0 | 0.818 |
| TF3 | GARNEFSKI N 2001 | 2 | 1163 | 0 | 0.811 |
| TF3 | GROSS JJ 2002 | 2 | 1079 | 0 | 0.809 |
| TF3 | COMPAS BE 2017 | 2 | 1366 | 0 | 0.799 |
| TF3 | SILK JS 2003 | 2 | 1476 | 0 | 0.774 |
| TF3 | NOLEN-HOEKSEMA S 2008 | 2 | 845 | 0 | 0.741 |
| TF3 | MCRAE K 2012 | 2 | 1094 | 0 | 0.736 |
| TF3 | GARNEFSKI N 2006 | 2 | 866 | 0 | 0.723 |
| TF3 | GROSS J. 2007 | 2 | 960 | 0 | 0.7 |
| TF3 | KESSLER RC 2005 | 2 | 766 | 0 | 0.604 |
| TF3 | AHMED SP 2015 | 2 | 801 | 0 | 0.548 |
| TF3 | MERIKANGAS KR 2010 | 2 | 540 | 0 | 0.419 |
| TF3 | MORRIS AS 2007 | 3 | 2043 | 95 | 0.933 |
| TF3 | THOMPSON R 1994 | 3 | 2120 | 34 | 0.973 |
| TF3 | EISENBERG N 1998 | 3 | 1025 | 0 | 0.733 |
| TF3 | ZEMAN J 2006 | 3 | 1087 | 0 | 0.668 |
| TF3 | MORRIS AS 2017 | 3 | 758 | 0 | 0.644 |
| TF3 | COLE PM 2004 | 3 | 852 | 0 | 0.581 |
| TF3 | SHIELDS A 1997 | 3 | 899 | 0 | 0.563 |
| TF3 | KIM J 2010 | 3 | 683 | 0 | 0.557 |
| TF3 | EISENBERG N 2010 | 3 | 784 | 0 | 0.533 |
