# Supplementary material for: From individual to relational: a scientometric mapping of emotion regulation in adolescence
Source: Front Psychol. 2026 Oct 8;17:1965549. doi: 10.3389/fpsyg.2026.1965549 (PMC13686872; doi:10.3389/fpsyg.2026.1965549)
Supplement: Supplementary file 2 [file Table_2.DOCX]

**Table S7.** BIBLIO checklist for reporting the present scientometric review, adapted from Montazeri et al. (2023).

| **Section** | **Item** | **Reporting requirement** | **Reported in manuscript** |
| --- | --- | --- | --- |
| **Title** | 1 | Identify the study as a bibliometric/scientometric review or analysis in the title. | Title – identified as a “Scientometric Mapping” |
| **Title** | 2 | Specify the main topic investigated and the time period covered by the review. | Topic: Title; Coverage period: Abstract and Section 2.2 |
| **Abstract** | 3 | Provide a structured or clearly organized summary of the background, methods, main findings, and conclusions, as applicable. | Abstract |
| **Introduction / Background** | 4 | Summarize the existing knowledge and provide the rationale for conducting the bibliometric review. | Section 1 |
| **Introduction / Background** | 5 | Clearly state the objectives or research questions of the review. | Section 1 |
| **Methods** | 6 | Describe the information sources and bibliographic databases used to retrieve the literature. | Section 2.2 |
| **Methods** | 7 | Report the search strategy, including search terms, date of search, language restrictions, and document-type criteria. | Section 2.2 |
| **Methods** | 8 | State the time period covered by the review and provide its rationale. | Sections 2.2 and 2.3.2 |
| **Methods** | 9 | Describe the eligibility criteria, including language, publication/document type, and temporal criteria. | Section 2.2 |
| **Methods** | 10 | Describe procedures used to refine and select the bibliographic dataset, including the removal of irrelevant, duplicate, or otherwise ineligible records, where applicable. | Section 2.2; Figure 1 |
| **Methods** | 11 | If applicable, describe any quality-assessment procedure used for the included publications. | Not applicable |
| **Methods** | 12 | Describe the methods used to summarize, analyze, synthesize, tabulate, and graphically represent the bibliographic data. | Sections 2.3, 2.3.1, and 2.3.2 |
| **Results** | 13 | Report the search and selection process, preferably through a flow diagram, together with the main descriptive bibliometric indicators (e.g., publication counts, document types, citations, sources, authors, and institutions, as applicable). | Figure 1; Section 3.1; Tables 1–5 |
| **Results** | 14 | Present relevant bibliometric maps, networks, temporal trends, or other graphical representations generated with appropriate analytical software. | Figures 2–9; Sections 3.2–3.4 |
| **Results** | 15 | Organize and summarize the findings using appropriate tables, thematic/conceptual categories, temporal organization, or other subdivisions relevant to the review objectives. | Sections 3.1–3.5; Tables 1–5; Figures 2–9 |
| **Results** | 16 | Synthesize the main findings and identify relevant patterns, gaps, or conceptual developments, where applicable. | Sections 3.2–3.5; Section 4 |
| **Discussion** | 17 | Summarize the principal findings in accessible and general terms. | Section 4 |
| **Discussion** | 18 | Interpret the findings consistently with the results and discuss possible explanations for the observed patterns, similarities, and differences. | Section 4 |
| **Discussion** | 19 | Discuss the strengths and limitations of the bibliometric review. | Section 4 |
| **Discussion** | 20 | Provide an overall interpretation of the findings in relation to the review objectives and discuss their potential implications. | Sections 4 and 5 |

**Note.** The checklist was completed according to the preliminary BIBLIO reporting guideline for bibliometric reviews proposed by Montazeri et al. (2023). Items not applicable to the present scientometric design are explicitly indicated.
